# Emerging AI approaches for cancer spatial omics

**DOI:** 10.1093/gigascience/giaf128

**Published:** 2025-10-16

**Authors:** Javad Noorbakhsh, Ali Foroughi pour, Jeffrey Chuang

**Affiliations:** The Jackson Laboratory for Genomic Medicine, Farmington, CT 06032, USA; St Jude Children’s Hospital, Memphis, TN 38105, USA; The Jackson Laboratory for Genomic Medicine, Farmington, CT 06032, USA; UCONN Health, Department of Genetics and Genome Sciences, Farmington, CT 06032, USA

**Keywords:** artificial intelligence, spatial transcriptomics, spatial proteomics, deep learning, foundation models, tissue biophysics

## Abstract

Technological breakthroughs in spatial omics and artificial intelligence (AI) have the potential to transform the understanding of cancer cells and the tumor microenvironment. Here we review the role of AI in spatial omics, discussing the current state-of-the-art and further needs to decipher cancer biology from large-scale spatial tissue data. An overarching challenge is the development of interpretable spatial AI models, an activity that demands not only improved data integration but also new conceptual frameworks. We discuss emerging paradigms—in particular, data-driven spatial AI, constraint-based spatial AI, and mechanistic spatial modeling—as well as the importance of integrating AI with hypothesis-driven strategies and model systems to realize the value of cancer spatial information.

## Background

Recent advances in highly multiplex spatially resolved omics (SRO), such as spatial transcriptomics and proteomics, have led to an explosion of studies on tissue spatial structure and its cellular underpinnings [1, 2]. Such approaches have the potential to revolutionize the histopathologic and molecular understanding of cancer. However, spatial data demand new analysis methods and concepts to address growing challenges in interpretability and reproducibility. Such challenges arise from the vastness of the data space spanning the intricate, yet largely undefined, spatial phenotypes within diverse tissue samples [3, 4]. Novel paradigms for biological discovery are needed to realize the translational value of these rich spatial resources.

SRO data are growing rapidly through imaging technologies, including stain-based (e.g., hematoxylin and eosin [H&E], immunohistochemistry [IHC]), molecular mass spectrometry (e.g., matrix-assisted laser desorption/ionization [MALDI]), transcriptomic (e.g., Visium, VisiumHD, Xenium, CosMx), and antibody-based proteomic (e.g., CODEX, CellDive) methods [5]. Low-plex stain-based methods have been widely used in clinical settings for decades. Mass spectrometry methods are versatile for measuring diverse molecular species, though with associated challenges in specificity [6]. Most prominently, spatial transcriptomic and proteomic methods have accelerated in the past few years, providing high-plex, highly specific quantifications of RNAs and proteins at resolutions of ∼0.25–100 microns [7]. These spatial approaches extend single-cell and bulk gene-profiling technologies that have been used widely in the past decade [5].

Due to the diversity and complexity of spatial phenomena within tissues, data-driven artificial intelligence (AI) approaches, in addition to mechanistic models, will be valuable to obtain insights from SRO data. Large-scale datasets are critical for AI-based data mining, and the scientific community will rely on spatial data being organized in computationally efficient, reusable, and standardized ways. Analogous organizational pressures arose during the high-throughput sequencing revolution, with cancer consortium projects (e.g., The Cancer Genome Atlas [TCGA], Pan-Cancer Analysis of Whole Genomes [PCAWG]) driving demand for standardization in datatypes (FASTQ, binary alignment map [BAM]) and genomic annotations (genes, variants, expression), centered on the goal of discovering driver mutations or expression states [8, 9]. More recent consortia (Human Cell Atlas [10], HuBMAP [11], Human Tumor Atlas Network [HTAN] [12], SenNet [13], etc.) and data aggregation initiatives (e.g., CROST [14] and STOmicsDB [15]) have been important for spatial omics data. However, the core goals of tissue spatial analysis have not yet been agreed upon. Despite the community’s extensive experience with bulk and dissociated sequencing and protein data, effective goal setting will require openness to new conceptual paradigms.

The most prevalent paradigm for spatial omics analysis has been to extend approaches from single-cell analysis—that is, first, to aggregate sequence (or protein) data for each cell and, second, to analyze how expression relates to cell location [16, 17]; however, this paradigm has inherent limitations. As an illustration, pathologists often make clinically valuable decisions from histology images, which contain no sequence data. Some cancer pathology evaluations may not even depend on cells, for example, relying on the morphology and density of blood vessels, the extracellular matrix, or necrotic cavities. Thus, cell-based approaches contain only part of the information valuable in tissue images. To determine the tissue features most relevant to patient outcomes, it will be vital to study not only the value of different data types but also how to best encode SRO data into useful data representations. The choice of data representation (e.g., cell-based [18], graph-based [19, 20], tile-based [21]) limits the biophysical processes that can be studied, but such limitations have so far been little investigated.

In the following sections, we discuss key conceptual challenges and possibilities for the understanding of spatial data, including the role that AI can play. While such topics pertain broadly to tissue biology, spatial profiling has special translational value for cancer. Spatial relationships are critical to treatment response, for example, by mediating the interactions of immune and cancer cells during immunotherapy. We conclude by discussing the need for tissue studies within perturbable model systems, which are needed to verify mechanistic understanding and pave the way for clinical translation.

## Main text

### Emerging analysis paradigms for cancer spatial AI

**Figure 1:**
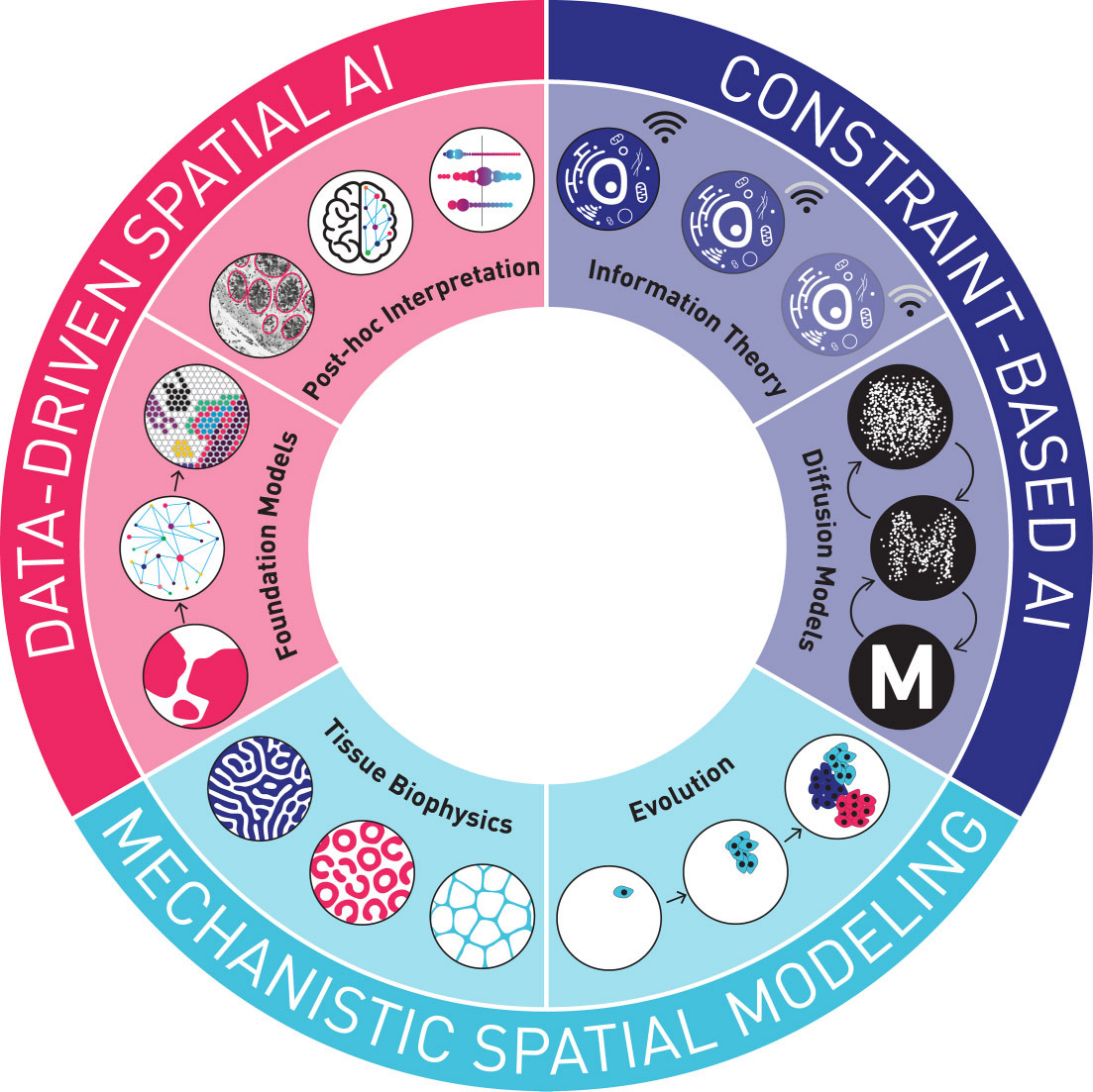
Emerging analysis paradigms for cancer spatial AI. DATA-DRIVEN SPATIAL AI is a paradigm that avoids strong assumptions about the underlying data. For example, Foundation Models can be trained on large-scale histopathology or spatial omic data to complete diverse tasks at the level of histopathology, cellular networks, or individual cells. Foundation models typically involve deep neural networks with abstract feature spaces, so Post Hoc Interpretation is needed to identify the structures in the tissue predictive of cancer outcomes. A second paradigm is CONSTRAINT-BASED SPATIAL AI, in which intuitive concepts are used to constrain model inference. Information Theory is an important framework to guide such models, as cellular interactions in cancer are constrained by information transfer. Diffusion Models are constraint-based AI models used widely in generative image AI and increasingly in tissue analysis, based on the presence of spatial hierarchies in images. MECHANISTIC SPATIAL MODELING is a paradigm based on hypothesis testing and mechanistic discovery. Tissue Biophysics approaches integrate spatial biophysical concepts (e.g., reaction diffusion, tissue mechanics) with empirical spatial omics observations. Evolution is fundamental to the development and treatment response of tumors, and new spatial omics-based approaches are improving the quantification and understanding of this process.

The field of spatial omics is in the early stages of establishing a hierarchy of goals, a prerequisite for the evaluation of data collection and analysis approaches. Nevertheless, several concepts have begun to emerge. Here we review approaches of growing importance, which we organize into 3 paradigms: Data-Driven Spatial AI, Constraint-Based Spatial AI, and Mechanistic Spatial Modeling (Fig. 1).

#### Data-driven spatial AI

Many recent spatial analysis algorithms have been data-driven (i.e., they seek to identify patterns in data without prespecifying biological hypotheses). Such approaches have the advantage of flexibility but can be difficult to interpret mechanistically, particularly when they use deep neural networks to embed biological data into abstract latent spaces. We consider 3 topics among the data-driven approaches: histopathology foundation models, spatial omic foundation models, and post hoc interpretation.

##### Histopathology foundation models

Histopathology foundation models have become a leading category of spatial machine learning models [22], typically involving deep neural network transformer architectures trained on whole-slide H&E-stained images (Fig. 1, Foundation Models). Foundation models are large machine learning models trained on vast datasets, enabling them to tackle diverse tasks [23]. For example, a single foundation model may enable image classification, segmentation, and annotation. In digital and computational pathology, these models have been trained on large H&E slide datasets, in some cases more than 1 million whole-slide images, and have demonstrated value for integration into diagnostic workflows [24, 25]. Such foundation models encode images into data representations, which can be used for disease classification, cell segmentation and outcome prediction. Other clinically driven image modalities, such as IHC, have also been studied using foundation models [26], but H&E models are the most well developed, due to the prevalent use of H&E by pathologists for clinical decisions.

Histopathology foundation models are based on deep neural network architectures applied to tiles within H&E whole-slide images. Slide representations are then computed by aggregating the behaviors of the tiles. Early foundation models were based on deep convolutional networks [27], but most recent models utilize self-supervised training with a vision transformer [28] backbone. One popular implementation is DINOv2 [29], which seeks to distinguish visual features without model fine-tuning. This architecture has been used in recent foundation models, including Virchow [25], UNI [30], and GPFM [31]. Histopathology foundation models have become progressively easier to use, for example, having been integrated into end-to-end workflows such as STAMP [32], which facilitates the preprocessing of whole-slide H&E images, model training, and evaluation in a single framework.

The wide success of large language models (LLMs), such as GPT [33], Bidirectional Encoder Representations from Transformers (BERT) [34], and LLaMA [35], has also sparked approaches to merge these with imaging tasks. A recent category of foundation models uses paired H&E images and text (e.g., CONCH [36], PathChat [37], and TITAN [38]). These approaches combine vision transformers and LLMs to generate integrated data representations and enable chat-based interrogation of histology images.

##### Spatial omic foundation models

Spatial omics data analysis has benefited from neural network approaches for many tasks (e.g., cell-type annotation, batch correction, resolution enhancement, clustering, spatially variable gene detection, dropout imputation, and ligand–receptor detection) [39], but general spatial omic foundation models are only beginning to be developed (Fig. 1, Foundation Models). It is instructive to compare with dissociated single-cell-based foundation models, which are simpler yet still growing rapidly. For example, a wave of tools leveraging transformers has emerged for single-cell deep learning [40]. A key innovation has been in the tokenization step, which specifies what aspects of the input data are important (e.g., gene identity, expression value, ranking, and metadata) [41]. For instance, in scGPT [42] tokens comprise genes, expression quantile orders, and experimental conditions. scBERT [43] uses gene2vec [44] to embed coexpression patterns into gene tokens, while binning the expression values. scGPT, scFoundation [45], and CellPLM [46] are foundation models whose resultant embeddings, whether fine-tuned or direct, can be used as inputs for various tasks. Other models include tGPT [47], xTrimoGene [48], TOSICA [49], and Geneformer [50], and others [40]. A caveat of these foundation models is that the available pretraining datasets have been much smaller than for H&Es and LLMs [40], so caution in accepting their outputs is warranted. Such models should improve as the field matures through improved data, curation, and architectures.

Techniques devised for single-cell RNA foundation models are beginning to be extended to spatial models. Such models also typically rely on transformer architectures trained on large datasets using self-supervision. A few of these models have been introduced as foundation models and can enable multiple downstream tasks. In particular, CellPLM [46] is a transformer encoder/decoder variational autoencoder (VAE) that can do cell clustering, denoising, imputation, and cell-type annotation, while Nicheformer [51] is a transformer model capable of label transfer and prediction of cellular neighborhood compositions. Both methods use a combination of spatial and dissociated single-cell RNA sequencing (scRNA-seq) as input, which potentially boosts their ability to transfer information across the 2 data modalities. Other models, such as SpaFormer [52] and stEnTrans [53], are transformer models trained on spatial transcriptomics data, which could potentially be utilized as foundational.

Foundation models for spatial proteomics are also a growing area. Spatial proteomic technologies have facilitated in-depth investigation of the tumor microenvironment, offering insights into tumor dynamics and its interactions with the immune system [54, 55]. Advances in multiplex techniques, such as CODEX [56, 57], IMC [58], and Cell Dive [59], have allowed simultaneous measurement of many proteins at subcellular resolution. Such data can enable direct modeling of biophysical processes, including fine interactions such as synapse formation between cells [60]. However, integrating protein data across experiments remains challenging due to variations in protein panels and measured antibody intensity distributions. These issues may be alleviated by encoding protein data into more robust representations (e.g., by nonnegative matrix factorization [61] or generative neural networks), which have also enabled powerful capabilities such as combinatorial protein signal decomposition [62, 63]. A promising recent method named KRONOS [64] addresses the issue of protein marker heterogeneity by an innovative tokenization approach. The authors pass the marker identity information as a secondary positional encoding and effectively treat all markers as equivalent otherwise. Their model outperformed existing foundation models that are trained on out-of-domain histology data or multichannel cell profiling images. Still, such encodings may create nonintuitive statistical artifacts, and broader research into proteomic foundation model development and their benchmarking is important to the further development of the field.

##### Post hoc interpretation

Despite their impressive capabilities, foundation models are often criticized as “black box” systems because their causal logic is not directly interpretable [65, 66]. The loss of interpretability arises from the embedding of image data into abstract latent spaces using deep neural networks. Interpretation of the genetic and cellular features associated with foundation model image embeddings therefore must be *post hoc* (i.e., based on data patterns rather than causal relationships among biological entities) (Fig. 1, Post Hoc Interpretation). This is in contrast with mechanistic models, which are formulated from experimentally controllable features such as genes or cells.

Some architectures, such as transformer models, provide their *post hoc* interpretability through the attention mechanism, which highlights what input features are focused on by individual attention heads within the neural network. However, interhead correlations are difficult to interpret, and attention weights do not uniquely translate to output feature importances [67–69]. Explainable AI (XAI) techniques like SHapley Additive exPlanations (SHAP) [70] and Local Interpretable Model-Agnostic Explanations (LIME) [71] address some of these limitations by attributing model predictions to individual features. SHAP uses a game-theoretic strategy, treating features as cooperative players contributing to the prediction, while LIME builds a simpler, explainable model through local perturbations of the original model around a data point of interest. In addition to model perturbation, data perturbation can be used to identify biologically plausible perturbations through context-guided data generation [72]. Additionally, methods inspired by physical entropy and thermodynamics have been proposed to identify optimal explanations for model embeddings [73]. These techniques can help capture spatial structures in images that are most indicative of outcomes. Other efforts have been made to develop interpretable models of features related to tissue organization. A useful concept is the functional tissue unit (FTU) [74], defined as the smallest multicellular tissue unit performing a specific function within its microenvironment that is replicated in a whole organ [75]. Repetitive FTUs can be captured by spatial frequency analysis of SRO data. For example, SpaGFT converts the SRO data into a graph and applies a graph Fourier transform to identify such features. This method can be implemented as an explainable regularizer for other machine learning models, improving their interpretability [76]. Such modular approaches based on FTUs, even if defined post hoc, are likely to be increasingly used in SRO analysis to demonstrate robustness and reusability of computational frameworks.

However, as technologies for proteomic plexity and throughput improve, deep learning and interpretability considerations will grow for these data types as well [63].

A limitation of post hoc interpretation approaches is that they reveal associations rather than causal relationships, making them more suited for hypothesis generation. These approaches can inadvertently attribute importance to spurious confounders and batch effects, so their use should be accompanied by batch correction and, ideally, validation on external datasets.

#### Constraint-based spatial AI

Alternative paradigms based on constraints on the SRO data representation can provide more interpretability than foundation models. For example, all tissue structures are constrained by biophysical processes, but the underlying cellular mechanisms are variable and complex. AI approaches that account for these constraints abstractly, without focusing on individual mechanisms, have grown. We discuss such approaches for cancer, focusing on those incorporating information theoretic constraints and those that mimic spatial constraints, notably image diffusion models.

##### Information theory–based constraints

Information theory is a mathematical framework for quantifying the flow, processing, and storage of information within systems [77], and it can be applied to guide spatial analysis in tumors. For example, information-theoretic concepts can be used to quantify limits on tissue heterogeneity, signaling dynamics, and other types of spatial organization, such as intratumoral immune infiltration and epithelial-to-mesenchymal transitions. Such approaches are helpful for understanding the bounds on inference for different models (Fig. 1, Information Theory).

Spatial information transfer in tissues has fundamental limits. This has been demonstrated for *Drosophila* embryos, in which morphogen gradients have been optimized by evolution to the physical limits of signal transduction [78]. Such evolutionary optimization likely extends to complex tissues, where information transfer is governed by physical and biological constraints that have been shaped by developmental processes. Viewing this as an information theory problem, with channel capacity and mutual information defining these constraints, provides a powerful analytical framework. In cancer, deviations from these optimal solutions may result in identifiable patterns in SRO data, revealing novel mechanisms driving cytokine, endocrine, and immune signaling [79, 80]. Quantifying signal propagation and interaction provides a way to understand collective cellular behaviors within cancer tissues driven by external or internal cues.

Spatial constraints on information transfer can motivate SRO dimensionality reduction approaches. For example, nonnegative matrix factorization (NMF) has been used to reduce glioma expression data into interpretable patterns of spatially coexpressed genes (i.e., “metaprograms”) [81], though it remains challenging to know what spatial scales are appropriate for such approaches. Alternatively, the information bottleneck (IB) method [82] offers an information-theoretic approach to attain the most compressed, lower-dimensional representation of a high-dimensional dataset that is maximally predictive of a desired outcome. bioIB [83] has developed this concept for scRNA-seq data to determine a set of genes (“meta-genes”) that are predictive of disease status or cell type. Extending such approaches to the spatial domain offers new opportunities for developing more robust SRO data analysis tools.

Extensions of IB have been applied to deep learning models, where the layers in the model are treated as an information channel, and IB is applied to each layer [84, 85]. These layers progressively represent a desired outcome, with each layer “forgetting” certain input details to better learn the output. Such methods could be extended to spatial data through architectural designs such as graph neural networks (GNNs), convolutional neural networks (CNNs), or vision transformers (ViTs) [28]. This type of strategy may elucidate how tissue-level information flows through molecular and spatial interactions, enabling the extraction of minimal yet predictive feature sets at multiple spatial scales. These minimal encodings could identify molecular signals and spatial arrangements for intercellular communication, revealing key length scales and structural patterns that govern tissue organization and behavior.

##### Image diffusion models

Diffusion models [86, 87] guided by theoretical frameworks that incorporate tissue-specific symmetries (e.g., repetitive structures, rotational and translational invariance) are a promising approach for spatial analysis (Fig. 1, Diffusion Models). These models have achieved significant advances in generative image AI, including widely used tools like DALL-E [88] and Stable Diffusion [89]. Diffusion models comprise an encoder that iteratively degrades an image’s data distribution structure through a diffusion-like process, followed by a decoder that reverses the process to reconstruct the image with progressive detail. Training such an encoder-decoder on image datasets yields a neural network able to generate images from noise [90]. Latent diffusion models [91] extend traditional diffusion approaches by first reducing the dimensionality of input data before applying the diffusion process to the compressed representation. This strategy enhances computational efficiency and has been integrated with transformers, forming diffusion transformers (DiTs) [92].

The large number of markers in spatial omics poses a challenge for diffusion models. Embedding data into a lower-dimensional space reduces noise, leverages marker correlations, and makes high-dimensional data more manageable. This latent diffusion model approach has been successfully applied to spatial transcriptomics data in methods such as stDiff [93] and SpaDiT [94], which utilize DiTs to impute missing genes, and may be useful for other high-dimensional omics types such as mass spectrometry as well. However, a key limitation of these models is their lack of spatial coordinate integration during training, which restricts their ability to fully capture and utilize spatial relationships. Some diffusion models integrate spatial information to better represent the unique characteristics of spatially resolved data. For example, DiffuST [95] employs latent diffusion models alongside graph autoencoders to resolve semantic inconsistencies across data modalities while capturing spatial relationships. SpatialDiffusion [96] incorporates spatial coordinates, gene expression, and cell-type information to predict unseen slices in 3-dimensional (3D) spatial transcriptomics, interpolating distributions from neighboring slices. Similarly, stMCDI [97] utilizes a GNN to encode spatial information, leveraging a diffusion model to impute missing data in spatial transcriptomics.

The success of diffusion models is rooted in statistical thermodynamics, as they leverage concepts such as phase transitions, symmetry breaking, and critical instabilities to achieve accurate image reconstruction [98], offering a theoretically supported approach for generating interpretable representations. While such models have been studied for images with few data channels, extending these principles to high-dimensional spatial omics presents a key challenge important for improving SRO data interpretation [99]. Furthermore, the iterative encoding process in diffusion models parallels the spatial hierarchies within images [100]. Investigation of this relationship may be valuable for improving interpretability. Another promising approach is context-constrained diffusion models [72], which can improve generation of biologically realistic data to facilitate interpretation of foundation models as well as provide augmented data during training.

#### Mechanistic spatial modeling

Tissue spatial profiling data are, essentially, measurements of 3D materials. In materials science, macroscopic behaviors are studied as mechanistically deriving from microscopic processes such as electromagnetic and molecular interactions. Likewise, tumors are impacted by microscopic biophysical processes such as chemical signaling and diffusion, resulting in contiguous region types (tumor, immune-infiltrated, necrotic, fibrotic, etc.) with internally coherent cell composition, cellular states, or extracellular structures. Some analysis approaches (e.g., GASTON [101]) leverage this coherence to identify region types. However, most current approaches are empirical rather than grounded in biophysical processes. Integration of physics-inspired concepts is therefore a promising direction, and there is a critical need for new AI models capable of mechanistic inference from SRO data. Below we discuss approaches using spatial data to learn biophysical and evolutionary processes within tumors.

##### Inference of tissue biophysics

Cellular dynamics within tissues can be viewed as a reaction-diffusion process (Fig. 1, Tissue Biophysics), where cell-intrinsic mechanisms and cell-to-cell communication occur simultaneously with diffusion. Computational models of cancer based on this perspective have been developed [102–104] but have been limited in the number of cell types covered and have relied on partial differential equations with many unknown parameters. However, for a hypothesized model of tissue architecture, the governing equations should be learnable if sufficient data are available. A recent category of inference method for learning equations from large datasets is physics-informed neural networks (PINNs) [105], which embed physical equations into neural networks. These methods require dynamical data for training, and they have been applied to scRNA data to predict cell state dynamics [106] and for RNA velocity inference [107]. So far, application of PINNs to tissues has been limited due to the scarcity of time-course data. However, as longitudinal SRO data improve, PINNs can provide a framework to infer interpretable physical processes and parameters from them. Nevertheless, PINNs can still be valuably applied to static data for diffusive systems at steady state or quasi-steady state, where timescales are sufficiently separated. Methods developed with this philosophy already exist and may be adaptable to PINNs. For example, HoloNet [108] is a graph neural network that uses ligand diffusion equations to infer ligand–receptor interactions from spatial transcriptomics data, and SpaCCC [109] integrates this into a transformer framework. These 2 methods overcome the limitations of time-course scarcity by stripping chemical diffusion and reaction equations from their dynamics and treating them as steady-state functional forms.

Drawing inspiration from these approaches, it may be possible to model cancer tissue as governed by multiple fields mediated by the spatial diffusion of molecules. An SRO PINN model could be built around cells acting as relays of such fields—each with its own transmission length scale, which could be inferred from gene or protein expression. These fields could describe, for example, molecular densities or mechanical forces, and cross-attention between them could reveal their interactions, such as ligand–receptor activation. Knowledge of chemical diffusion may be directly incorporated into PINN architecture. For example, multilayer perceptron modules could be used to link gene expression to signal concentration or diffusion rate, while convolution-like kernels could approximate the diffusion process across different length scales. This approach could provide a framework to quantify the spatial dynamics of immune, stromal, and cancer cell populations, as well as their modulation by host factors.

While most neural networks rely on multilayer perceptrons (MLPs), which are feed-forward networks with learnable linear edges and fixed nonlinear nodes, Kolmogorov–Arnold networks (KANs) [110] take the opposite approach, learning nonlinear functions on edges while keeping nodes linear. This design allows KANs to directly infer subfunctional components of a global function, making them potentially more interpretable. Also, although similar to PINNs in their ability to infer system dynamics, KANs are not limited to dynamic data. KANs integrated with convolutional neural networks have been successfully used in remote sensing applications [111] involving geographic spatial profiles at hundreds of light frequencies. Such data are analogous to spatial omics data in their high number of channels and environment-influenced spatial relationships. Thus, KANs may be effective for interpreting how groups of biomarkers and cell types form structural phenotypes within tissues, using the mathematical functions inferred along KAN edges.

Physical processes such as diffusion can also be qualitatively incorporated into neural networks. For instance, sepal [112] is a spatial transcriptomics analysis tool that assumes that transcripts diffuse in the environment according to a fixed diffusion rate. It then ranks the genes by the time required to reach homogeneity. Although in this method, the diffusion process is not physically observed, this approach is nevertheless able to determine spatial structures and their related gene families.

Tissue mechanical properties such as stiffness, adhesion, and viscosity are also important to cancer. These impact tumor invasiveness [113–115], drug diffusion, and angiogenesis, likely mediated by the impact of mechanical properties on cellular polarity, membrane rigidity, and cell migration [116, 117]. SRO measurements have the potential to reveal mechanical parameters. For example, spatial transcriptomics data have been used to infer tissue stiffness by solving the equations of surface tension from segmented cell membranes [118]. Although the use of cancer SRO data for tissue mechanics studies has been uncommon, the substantial literature on cancer tissue biophysics [119, 120] suggests that such approaches have untapped potential [121].

##### Evolution

Evolution has been extensively used to interpret tumor formation, heterogeneity, treatment response, and resistance (Fig. 1, Evolution). Many studies have incorporated scRNA-seq data toward understanding cancer evolution [122], and analogous spatial transcriptomics–based analysis are growing [123]. Because evolution is classically defined by genotypes, a common use of SRO data has been to optimize spatial genotype calling. Methods like InferCNV [124], which was developed to infer copy number variations (CNVs) from scRNA-seq data, are now regularly applied to spatial transcriptomics data [125] to identify local CNV-defined genotypes. Analogously, transformer-based CNV-calling methods like CoT [126], which has been used for genome-wide denoising of single-cell DNA sequencing (scDNA-seq), could be adapted for spatial DNA sequencing. Integrating spatial continuity into these models can improve subclonal phylogeography inference. CalicoST [127] does this, including inference of allele-specific copy numbers, and it has been used to identify subclonal heterogeneity as well as oncogenic and metabolic activity in HTAN datasets [123]. Currently, CalicoST does not leverage long-range spatial correlations, focusing on spot neighborhoods. However, long-range correlations could arise in images from geographic selection pressures within the tumor or due to the limited ability of 2-dimensional (2D) images to represent 3D spatial processes. Improved attention-based networks will be valuable to better capture these effects. PINNs have also been applied to capture tumor growth dynamics [128]. Combining these methods with genomic-based methods may improve phylogeography inference from SRO data.

A broader consideration for tumor evolution is that epigenetic and morphological changes occur together with genotype evolution. For example, epigenetic shifts in tumor cells are important to treatment resistance [125]. Therefore, simultaneous integration of spatial gene expression and phylogenetics in a single network would be valuable. This could be achieved by feeding expression and genotype matrices into a unified tokenization scheme. Tissue morphology, as characterized by H&E and analyzed via foundation models, has also been shown to predict some tumor expression states and genotypes [129, 130]. This suggests that it may be possible to improve inference of tumor evolutionary processes by embedding local genomic data together with the tissue morphology and transcriptional gradient context.

### Data considerations

#### Data integration

Cancer multimodal spatial data are diverse [123], creating data integration opportunities and challenges. At one end, H&E data are widely available but low plex. On the other end, spatial omics datasets are scarce but can have thousands of markers. It remains poorly understood what information is common across modalities and what is modality-specific. To benefit from spatial data integration, technical considerations must be addressed. Different modalities require distinct scaling, standardization, and filtering to avoid biasing downstream models. Image registration across modalities is challenging due to differences in data formats, mismatched markers, resolutions, sample preparation, tissue size, and batch effects [131]. Measurement noise, including technical and biological variability, can complicate data interpretation [132]. Spatial RNA can be affected by platform-specific dropout and probeset limitations [133].

Nevertheless, spatial data integration is already benefiting cancer research. For example, cancer H&E images have been shown to predict spatial expression–identified patterns, including immune infiltration and drug-induced persistence [21, 125, 134]. Pipelines are available to ease H&E/spatial transcriptomic integration for cancer, such as STQ [135]. Other general integration methods will also be useful for cancer. For example, SpatialGlue [136] integrates spatial transcriptomics, proteomics, and epigenomics data for domain identification. Architectures based on single-cell integration frameworks such as scGPT [42] and scMoFormer [137] provide a model for expanding spatial data integration. COVET [138] uses localized expression covariances to encode cellular niches in spatial transcriptomics data. ENVI [138] is a variational autoencoder that integrates with COVET to simultaneously infer missing genes in spatial transcriptomics data inferred from dissociated single-cell data, while assigning niche information to dissociated cells using spatial data. Approaches based on large-scale H&E integration with expression data are also of growing value [139]. For example, OmiCLIP [140] encodes highly expressed genes from local spots into sentences, then uses vision-language deep learning theory to build an H&E-omics foundation model.

Different spatial omics modalities from the same tissue often have mismatched resolution, complicating integration. Heterogeneous GNNs [141], which assign modality-specific attributes to nodes, have been used to study the tumor microenvironment in spatial transcriptomics data [142] and could potentially support multiresolution integration by linking nodes (e.g., cells or spots) by spatial proximity. Similarly, multiresolution transformers have shown success in histopathology [143] by jointly processing images at multiple zoom scales. A similar strategy could be adapted for spatial omics integration by treating each resolution as a distinct modality.

Platform-specific noise (e.g., spatial RNA dropout) further complicates integration. Methods adapted from single-cell analysis can help mitigate this issue. For example, LLOKI [144] combines neighborhood and distributional similarity and pretrained single-cell foundation models to reduce expression sparsity and correct batch effects across datasets. Divergent marker panels, particularly in spatial proteomics, also impede integration. This can be mitigated by recognizing that phenotypes often recur even when exact markers differ. Integration with other data types (e.g., H&E) and marker-agnostic encoding methods [64] can be effective as well.

#### Model benchmarking

Rapid progress in digital pathology foundation models makes rigorous benchmarking essential. Current *de facto* H&E benchmarks, such as TCGA subtype classification and molecular prediction [30, 145], largely reflect historical data availability rather than a coordinated community-driven effort aimed at biological discovery and translational impact [146]. To close this gap, the field needs community initiatives to curate agreed-upon public datasets and standardize metrics. While clinically grounded datasets and benchmarks are emerging [147, 148], broad institutional momentum remains limited; nonetheless, we anticipate increasing standardization in the coming years.

In the SRO domain, the need is even more acute, as limited large-scale datasets and rapidly evolving assays make benchmarking ambiguous. Existing benchmarks mostly target unsupervised tasks (e.g., spatial domain identification) or self-supervised objectives (e.g., imputing missing genes), or they rely on expert-annotated datasets that are small and domain-specific [39]. Where labels exist, they are typically intratissue (e.g., cell-type annotations) rather than cross-sample, and patient-level annotation is scarce. These benchmarks are valuable for research, but to build parallels to the H&E AI models would require clinically grounded benchmarks built on large-scale patient-level annotations. Organizing consortia to generate clinically annotated, patient-derived spatial omics datasets is essential to establish relevant ground truth and enable clinically meaningful benchmarking.

#### Mechanism-driven data generation

Spatial data have tremendous potential for improving prediction of clinical outcomes (e.g., [149]) while simultaneously suggesting new mechanisms. However, post hoc interpretation approaches conflict with hypothesis-oriented standards for mechanistic discovery. For example, cancer clinical H&E samples have been studied extensively by foundation model approaches, but it remains unclear how to ascertain causal relationships from them. Patient institutional review board considerations also limit the types of clinical samples that can be obtained. Such issues will constrain human SRO-based foundation model approaches, even if large cancer datasets can be amassed [150]. To verify causal mechanisms, it will be important to purposefully generate spatial data for mechanistic discovery.

Mice are the canonical mammalian model organism, making them ideal for the study of cancer spatial mechanisms in a tissue context. The ease of mouse genetic engineering and organismal perturbation has a long history of enabling hypothesis-based discoveries of oncogenes, tumor suppressors, metastatic processes, tumor environmental interactions, and effects of aging [151]. Sophisticated mouse population genetic systems, such as the Diversity Outbred and Collaborative Cross [152], have also revealed genes important to cancer mechanisms [153] and quantitative trait loci predictive of cell morphology [154]. Dynamic SRO data can be generated more easily in mice than from clinical samples, a key need for the training of PINNs. Stated simply, mice and other organismal models enable hypothesis-driven science on tissues, addressing the central challenge of post hoc mechanistic interpretation.

To realize the value of mouse and other animal model SRO cancer studies, such knowledge must be transferable to human. Identification of orthologous behaviors between mouse and human has not yet been well quantified with SRO data, although projects such as the Cellular Senescence Network (SenNet) [13] are performing spatial profiling of tissues across some matched mouse and human organs. Methods for the scRNA version of this problem are being actively developed. For example, CAME [155] aligns scRNA-seq data across species and enables the transfer of cell-type labels, which it accomplishes via a graph neural network that integrates a gene–gene graph of homologous genes and a cell–cell graph of transcriptionally similar cells. BrainAlign [156] extends this idea to spatial transcriptomics by adding a graph linking spatially proximal spots on the tissue. BrainAlign can align human and mouse brain tissue as well as identify conserved and species-specific gene expression patterns. Existing SRO transformer models may also be developed for human and mouse SRO comparisons. For example, Nicheformer [51] uses a unified gene-based tokenizer for human and mouse spatial transcriptomics and scRNA-seq, producing embeddings for tasks such as spatial label prediction. However, it does not use spatial coordinates as input. In contrast, SpaFormer [52] uses a cell-based tokenizer and explicitly encodes positional information to impute missing spatial transcriptomics data. A combined architecture with multispecies tokenizers and explicit positional encodings could better align human and mouse SRO data. All these approaches will require further development of expert annotated SRO sets in mouse and human to train cross-species spatial aligners. Despite evolutionary similarities, human and mouse may diverge in many aspects of tissue architecture, including fine immune microenvironmental organization and gene expression dynamics [157]. Computational mapping of tissue architecture across the 2 species is a broad challenge whose solution would improve the translational use of mouse models for human cancer research.

### Conclusions

The field of spatial omics is expanding rapidly in cancer research due to the critical importance of location-dependent interactions of cells within the tumor microenvironment. Cancer spatial omics datasets are high-dimensional and diverse, necessitating improved analytical paradigms. We have described 3 major paradigms for the development of the field: data-driven spatial AI, constraint-based spatial AI, and mechanistic spatial modeling. We have also reviewed key additional considerations in data integration and mechanism-driven data generation.

While the scale and complexity of SRO data create strong demand for AI-based approaches, deep neural networks have only post hoc interpretability compared to classical hypothesis-oriented approaches. Among the 3 major paradigms, deep neural network–based data-driven approaches, such as foundation models, are the least interpretable or mechanistic. Constraint-based spatial AI, such as image diffusion models, improves on this by incorporating biologically reasonable constraints into the underlying neural networks. Mechanistic models are the most interpretable, as they are constructed based on experimentally perturbable entities such as cells or genes. PINNs are a particularly promising approach for mechanistic modeling, as they combine explicit mathematical modeling of perturbable entities with data-driven inference amenable to SRO data. This provides a framework to jointly study biophysical and cell biological processes within tissues. Such joint investigation is essential to the underlying spatial processes of cell motility, signaling, replication, and evolution within the tumor microenvironment.

A general challenge for spatial omics foundation models is validation. Although tests such as tumor-type classification are commonly accepted for H&E foundation model benchmarking, spatial omics foundation model benchmarking is not yet standardized. To be useful, SRO foundation models should be able to distinguish fine microenvironments within tumors, a more complex task that is also inherently multiscale. Thus, SRO foundation model benchmarking will require community agreement on standardized evaluation tasks at multiple spatial scales. Alternative statistical models may be valuable in circumventing these challenges (e.g., constraint-based statistical models can be more interpretable and require less training data than deep learning counterparts) [158]. Models based on message passing are a canonical example, where information of neighboring cells is aggregated to represent the cellular composition and spatial relations [159]. These models share similarities with GNNs but require less training data. Interestingly, recent studies have combined GNNs with message-passing techniques to generate improved embeddings given small data [160–162].

While spatial data are growing for clinical cancer samples, clinical data are restricted by regulatory and collection limitations. These restrictions make it difficult to generate the perturbative data important for hypothesis-based science or the time courses important for training of PINNs. Mouse models can address this problem, as biophysical and genetic perturbations are possible with mice, and time-course data are easier to generate with mice than in the clinic. Still, mice may respond differently to treatment than patients, and SRO-based models trained on mice will likely need to be fine-tuned on human data. Eventually, curated repositories that integrate human and mouse SRO cancer tissue data will be vital for the field. These will enable improved identification and validation of functional tissue units, reusable data analysis, and more precise delineation of the spatial processes essential to cancer marker identification, drug targeting, and clinical translation.

## Abbreviations

AI: artificial intelligence; BAM: binary alignment map; BERT: Bidirectional Encoder Representations from Transformers; CNN: convolutional neural network; CNV: copy number variation; DiT: diffusion transformer; FTU: functional tissue unit; GNN: graph neural network; H&E: hematoxylin and eosin; HTAN: Human Tumor Atlas Network; IB: information bottleneck; IHC: immunohistochemistry; KAN: Kolmogorov–Arnold network; LIME: Local Interpretable Model-Agnostic Explanations; LLM: large language model; MALDI: matrix-assisted laser desorption/ionization; MLP: multilayer perceptron; NMF: nonnegative matrix factorization; PCAWG: Pan-Cancer Analysis of Whole Genomes; PINN: physics-informed neural network; RNA-seq: RNA sequencing; scDNA-seq: single-cell DNA sequencing; scRNA-seq: single-cell RNA sequencing; SenNet: The Cellular Senescence Network; SHAP: SHapley Additive exPlanations; SRO: spatially resolved omics; TCGA: The Cancer Genome Atlas; VAE: variational autoencoder; ViT: vision transformer; XAI: explainable artificial intelligence.

## Supplementary Material

giaf128_Authors_Response_To_Reviewer_Comments_Original_Submission

giaf128_GIGA-D-25-00248_Original_Submission

giaf128_GIGA-D-25-00248_Revision_1

giaf128_Reviewer_1_Report_Original_Submission Carolina Wählby -- 7/11/2025

giaf128_Reviewer_2_Report_Original_Submission Yingcheng Wu -- 8/8/2025

## Data Availability

Not applicable
