# Supplementary material for: Emerging AI approaches for cancer spatial omics
Source: Gigascience. 2025 Oct 16;14:giaf128. doi: 10.1093/gigascience/giaf128 (PMC12612624; doi:10.1093/gigascience/giaf128)
Supplement: giaf128_GIGA-D-25-00248_Revision_1 [file giaf128_giga-d-25-00248_revision_1.pdf]

GigaScience

Emerging AI Approaches for Cancer Spatial Omics

--Manuscript Draft--

**Manuscript Number:**	GIGA-D-25-00248R1
**Full Title:**	Emerging AI Approaches for Cancer Spatial Omics
**Article Type:**	Review
**Funding Information:**	NIH (R01 CA230031) — Dr Jeffrey H Chuang<br>NIH (U54 AG075941) — Dr Jeffrey H Chuang<br>NIH (P30 CA034196) — Dr Jeffrey H Chuang
**Abstract:**	Technological breakthroughs in spatial omics and artificial intelligence (AI) have the potential to transform the understanding of cancer cells and the tumor microenvironment. Here we review the role of AI in spatial omics, discussing the current state-of-the-art and further needs to decipher cancer biology from large-scale spatial tissue data. An overarching challenge is the development of interpretable spatial AI models, an activity which demands not only improved data integration, but also new conceptual frameworks. We discuss emerging paradigms – in particular data-driven spatial AI, constraint-based spatial AI, and mechanistic spatial modeling -- as well as the importance of integrating AI with hypothesis-driven strategies and model systems to realize the value of cancer spatial information.
**Corresponding Author:**	Jeffrey Chuang<br>Jackson Laboratory - Farmington<br>Farmington, CT UNITED STATES
**Corresponding Author Secondary Information:**	
**Corresponding Author's Institution:**	Jackson Laboratory - Farmington
**Corresponding Author's Secondary Institution:**	
**First Author:**	Javad Noorbakhsh, Ph.D
**First Author Secondary Information:**	
**Order of Authors:**	Javad Noorbakhsh, Ph.D<br>Ali Foroughi pour, Ph.D<br>Jeffrey H Chuang, Ph.D
**Order of Authors Secondary Information:**	
**Response to Reviewers:**	Note: We have provided a file describing all responses, described as "Personal Cover." The content is identical to the material below, but the responses are marked in red to more clearly separate the reviewer comments and responses.<br><br>Dear GigaScience,<br>We would like to thank the reviewers and editor for generously taking the time to consider this manuscript. The constructive feedback has helped us improve the work. Point-by-point responses to each of the reviewer comments are provided below.<br><br>Best regards,<br>Jeff Chuang<br><br>Reviewer reports:<br>Reviewer #1.1: This is a very nice review providing a new and original angle to the present stat e of combining AI-based analysis approaches with spatially resolved omics methods (brrimarily proteomics and transcriptomics). The review also points to

Powered by Editorial Manager® and ProduXion Manager® from Aries Systems Corporation

new directions of development, and is truly inspirational in its outlooks and future perspectives, which is appreciated. The text is well-written and easy to follow, though at points a bit brief in methods descriptions, as can be expected from this type of broad review paper. I was missing references in the introduction, feeling that many of the statements would have benefitted from pointing to e.g. other review papers in the field. Adding more references to the introduction would improve the text and also make the review more useful for an audience without expert knowledge in spatially resolved omics.

Response: We thank the reviewer for their comment and agree that sufficient references were lacking from our introduction. We added several references to the introduction of the paper most of which are review papers. We also noticed that in other chapters, concepts such as diffusion models and information theory lacked references. We have added references for those as well.

2. Abbreviations are thoroughly described apart from 'patient IRB', which should be defined, or perhaps re-phrased to not use an abbreviation.

Response: We added the IRB abbreviation to the end of the manuscript.

Reviewer #2.1a: The manuscript outlines three emerging paradigms (data-driven, constraint-based, and mechanistic spatial AI) but lacks a rigorous critical analysis of their practical limitations in cancer research. For data-driven foundation models, while post-hoc interpretation methods (e.g., SHAP, LIME) are mentioned, the review does not sufficiently address their inherent weaknesses—such as the inability to establish causal relationships or the risk of overfitting to batch effects in heterogeneous spatial omics datasets.

Response: We thank the reviewer for their comment. We agree that the way we wrote the text could confuse the reader about the link between the post-hoc interpretation and causal analysis. We have added the following paragraph to clarify this point:

“A limitation of post-hoc interpretation approaches is that they reveal associations rather than causal relationships, making them more suited for hypothesis generation. These approaches can inadvertently attribute importance to spurious confounders and batch effects, so their use should be accompanied by batch correction, and ideally, validation on external datasets.”

1b: Similarly, constraint-based models (e.g., diffusion models) are noted for integrating spatial hierarchies, but there is little discussion of how their performance scales with high-dimensional spatial proteomics data (e.g., >1000 markers) or their susceptibility to artifacts from variable antibody panels.

Response: Current antibody-based proteomics data tends to be low-dimensional (on the order of 10-100 protein). High-dimensional protein data (>1000 markers) of the sort the reviewer mentions tend to be masspec-based (e.g. MALDI), and such data have challenges similar to spatial transcriptomics. We have now mentioned these challenges for high dimensional spatial omics data in the text and proposed latent diffusion models as a solution (e.g. DiTs). Some methods using this approach are already mentioned in the text (e.g. SpaDiT, stDiff, DiffuST). However, we recognize that the text may not have been clear enough in linking these ideas. We added the following to the text:
“The large number of markers in spatial omics poses a challenge for diffusion models. Embedding data into a lower dimensional space reduces noise, leverages marker correlations, and makes high-dimensional data more manageable. This latent diffusion model approach has been successfully applied to spatial transcriptomics data in methods such as stDiff [1] and SpaDiT [2] which utilize DiTs to impute missing genes, and may be useful for other high dimensional omics types such as mass spectrometry as well. “

1c: Mechanistic models like PINNs are highlighted, yet the manuscript overlooks critical barriers such as the scarcity of longitudinal spatial data in clinical cancer samples, which limits their training and validation. A more balanced assessment of these limitations is essential for readers to gauge the current translational readiness of each

Powered by Editorial Manager® and ProduXion Manager® from Aries Systems Corporation

paradigm.

Response: We thank the reviewer for this comment. We note that some of the limitations of PINNs are mentioned in the text already:

“These methods require dynamical data for training, and they have been applied to scRNA data to predict cell state dynamics [3] and for RNA velocity inference [4]. So far, application of PINNs to tissues has been limited due to scarcity of time-course data. However, as longitudinal SRO data improve, PINNs can provide a framework to infer interpretable physical processes and parameters from them.”

However, even without time-series data these frameworks are valuable, a point which may not have been sufficiently clear. We therefore added the following sentences to the text:
“Nevertheless, PINNs can still be valuably applied to static data for diffusive systems at steady-state or quasi-steady state, where timescales are sufficiently separated. Methods developed with this philosophy already exist and may be adaptable to PINNs.”
This provides more context for the prior text, which follows immediately afterward:
“For example, HoloNet [5] is a graph neural network that uses ligand diffusion equations to infer ligand-receptor interactions from spatial transcriptomics data, and SpaCCC [6] integrates this into a transformer framework. These two methods overcome the limitations of time-course scarcity by stripping chemical diffusion and reaction equations from their dynamics and treating them as steady-state functional forms.”

2a: The review emphasizes the importance of multi-modal data integration (e.g., H&E, spatial transcriptomics, proteomics) but fails to address key technical bottlenecks in achieving robust integration. For instance, while methods like SpatialGlue and OmiCLIP are cited, there is little discussion of how to resolve fundamental inconsistencies: varying spatial resolutions (0.25-100 microns across technologies)...

Response: We added the following paragraph to cover the issue of ‘varying spatial resolutions’ and proposed some approaches to address it:
“Different spatial omics modalities from the same tissue often have mismatched resolution, complicating integration. Heterogenous GNNs [7], which assign modality-specific attributes to nodes, have been used to study the tumor microenvironment in spatial transcriptomics data [8] and could potentially support multi-resolution integration by linking nodes (e.g. cells or spots) by spatial proximity. Similarly, multi-resolution transformers have shown success in histopathology [9] by jointly processing images at multiple zoom scales. A similar strategy could be adapted for spatial omics integration by treating each resolution as a distinct modality.”

2b: ...platform-specific noise (e.g., dropout in spatial RNA), and divergent marker panels in proteomics. This gap hinders understanding of the current feasibility of holistic spatial profiling.

Response: We agree this is an important topic. We refer the reviewer to the ‘Spatial omics foundation models’ section which we believe addresses their concern about divergent marker panels:
“However, integrating protein data across experiments remains challenging due to variations in protein panels and measured antibody intensity distributions. These issues may be alleviated by encoding protein data into more robust representations, e.g. by non-negative matrix factorization [10] or generative neural networks, which have also enabled powerful capabilities such as combinatorial protein signal decomposition [11] [12]. A promising recent method named KRONOS [13] addresses the issue of protein marker heterogeneity by an innovative tokenization approach.”

Still, as the reviewer correctly points out, this topic could also be perceived as a data integration challenge. We added the following to the ‘data integration’ section to directly address this issue:
“Platform-specific noise (e.g. spatial RNA dropout) further complicates integration. Methods adapted from single-cell analysis can help mitigate this issue. For example, LLOKI [14] combines neighborhood and distributional similarity and pretrained single-

cell foundation models to reduce expression sparsity and correct batch effects across datasets. Divergent marker panels, particularly in spatial proteomics, also impede integration. This can be mitigated by recognizing that phenotypes often recur even when exact markers differ. Integration with other data types (e.g. H&E) and marker-agnostic encoding methods [13] can be effective as well. "

2c: Additionally, despite noting mouse models as critical for mechanistic validation, the manuscript underdeveloped the challenge of cross-species translation. While BrainAlign and Nicheformer are mentioned, there is insufficient analysis of how species-specific differences in tissue architecture (e.g., immune microenvironment organization) or gene expression dynamics might invalidate model transferability. Without addressing these, the review risks overstating the practical utility of current integration frameworks.

Response: We thank the reviewer for their comment and added the following caveat to the text:
"Despite evolutionary similarities, human and mouse may diverge in many aspects of tissue architecture including fine immune microenvironmental organization and gene expression dynamics [15]. Computational mapping of tissue architecture across the two species is a broad challenge whose solution would improve the translational use of mouse models for human cancer research."

3. The manuscript acknowledges that spatial omics foundation models lack standardized benchmarking but does not sufficiently elaborate on this critical gap. For histopathology foundation models, tumor-type classification is noted as a common benchmark, but the review does not propose how to evaluate more complex tasks essential for cancer research—such as distinguishing fine-grained tumor microenvironments (e.g., immune-hot vs. immune-excluded regions) or predicting treatment response from spatial patterns. Similarly, for spatial transcriptomics/proteomics models, there is no discussion of reference datasets, gold-standard metrics (beyond clustering or imputation accuracy), or inter-lab reproducibility challenges. This omission is problematic because without agreed-upon benchmarks, progress in the field risks being fragmented and difficult to compare. The review would benefit from proposing a roadmap for community-driven standardization, including multi-scale evaluation tasks and validation against clinical endpoints (e.g., patient survival).

Response: We added a new subsection titled 'Model benchmarking' to the 'Data consideration' section to address this issue:
"Rapid progress in digital pathology foundation models make rigorous benchmarking essential. Current de facto H&E benchmarks, such as TCGA subtype classification and molecular prediction [16], [17], largely reflect historical data availability rather than a coordinated community-driven effort aimed at biological discovery and translational impact [18]. To close this gap, the field needs community initiatives to curate agreed-upon public datasets and standardize metrics. While clinically grounded datasets and benchmarks are emerging [19], [20], broad institutional momentum remains limited; nonetheless, we anticipate increasing standardization in the coming years.
In the SRO domain the need is even more acute, as limited large-scale datasets and rapidly evolving assays make benchmarking ambiguous. Existing benchmarks mostly target unsupervised tasks (e.g. spatial domain identification) or self-supervised objectives (e.g. imputing missing genes), or rely on expert-annotated datasets that are small and domain-specific [21]. Where labels exist, they are typically intra-tissue (e.g. cell type annotations) rather than cross-sample, and patient-level annotation is scarce. These benchmarks are valuable for research, but to build parallels to the H&E AI models would require clinically grounded benchmarks built on large-scale patient-level annotations. Organizing consortia to generate clinically annotated, patient-derived spatial omics datasets is essential to establish relevant ground truth and enable clinically meaningful benchmarking."

References
[1]"stDiff: a diffusion model for imputing spatial transcriptomics through single-cell transcriptomics | Briefings in Bioinformatics | Oxford Academic." Accessed: Oct. 02, 2024. [Online]. Available: https://academic.oup.com/bib/article/25/3/bbae171/7646375
[2]X. Li, F. Zhu, and W. Min, "SpaDiT: Diffusion Transformer for Spatial Gene

	Expression Prediction using scRNA-seq," July 18, 2024, arXiv: arXiv:2407.13182. doi: 10.48550/arXiv.2407.13182.<br>[3]Q. Jiang and L. Wan, "A physics-informed neural SDE network for learning cellular dynamics from time-series scRNA-seq data," Bioinformatics, vol. 40, no. Supplement_2, pp. ii120–ii127, Sept. 2024, doi: 10.1093/bioinformatics/btae400.<br>[4]I. K. Boudjelthia, S. Milite, N. E. Kazwini, Y. Huang, A. Sottoriva, and G. Sanguinetti, "NeuroVelo: interpretable learning of temporal cellular dynamics from single-cell data," June 10, 2024, bioRxiv. doi: 10.1101/2023.11.17.567500.<br>[5]H. Li et al., "Decoding functional cell–cell communication events by multi-view graph learning on spatial transcriptomics," Brief. Bioinform., vol. 24, no. 6, p. bbad359, Nov. 2023, doi: 10.1093/bib/bbad359.<br>[6]B. Ji, L. Xu, and S. Peng, "SpaCCC: Large language model-based cell-cell communication inference for spatially resolved transcriptomic data," Feb. 23, 2024, bioRxiv. doi: 10.1101/2024.02.21.581369.<br>[7]C. Zhang, D. Song, C. Huang, A. Swami, and N. V. Chawla, "Heterogeneous Graph Neural Network," in Proceedings of the 25th ACM SIGKDD International Conference on Knowledge Discovery & Data Mining, in KDD '19. New York, NY, USA: Association for Computing Machinery, July 2019, pp. 793–803. doi: 10.1145/3292500.3330961.<br>[8]C. Zuo, J. Xia, and L. Chen, "Dissecting tumor microenvironment from spatially resolved transcriptomics data by heterogeneous graph learning," Nat. Commun., vol. 15, no. 1, p. 5057, June 2024, doi: 10.1038/s41467-024-49171-7.<br>[9]"[2206.02647] Scaling Vision Transformers to Gigapixel Images via Hierarchical Self-Supervised Learning." Accessed: Aug. 22, 2025. [Online]. Available: https://arxiv.org/abs/2206.02647<br>[10]A. F. Pour et al., "Prediction of Outcome from Spatial Protein Profiling of Triple-Negative Breast Cancers," Apr. 18, 2025, bioRxiv. doi: 10.1101/2025.04.18.649541.<br>[11]R. Ben-Uri et al., "High-dimensional imaging using combinatorial channel multiplexing and deep learning," Nat. Biotechnol., pp. 1–14, Mar. 2025, doi: 10.1038/s41587-025-02585-0.<br>[12]S. Ayub, H. W. Jackson, A. Selega, and K. R. Campbell, "Multi-view deep learning of highly multiplexed imaging data improves association of cell states with clinical outcomes," Mar. 17, 2025, bioRxiv. doi: 10.1101/2025.03.14.643377.<br>[13]M. Shaban et al., "A Foundation Model for Spatial Proteomics," June 05, 2025, arXiv: arXiv:2506.03373. doi: 10.48550/arXiv.2506.03373.<br>[14]E. Haber, A. Deshpande, J. Ma, and S. Krieger, "Unified integration of spatial transcriptomics across platforms," Apr. 21, 2025, bioRxiv. doi: 10.1101/2025.03.31.646238.<br>[15]T. Shay et al., "Conservation and divergence in the transcriptional programs of the human and mouse immune systems," Proc. Natl. Acad. Sci., vol. 110, no. 8, pp. 2946–2951, Feb. 2013, doi: 10.1073/pnas.1222738110.<br>[16]R. J. Chen et al., "Towards a general-purpose foundation model for computational pathology," Nat. Med., vol. 30, no. 3, pp. 850–862, Mar. 2024, doi: 10.1038/s41591-024-02857-3.<br>[17]H. Xu et al., "A whole-slide foundation model for digital pathology from real-world data," Nature, vol. 630, no. 8015, pp. 181–188, June 2024, doi: 10.1038/s41586-024-07441-w.<br>[18]F. Mahmood, "A benchmarking crisis in biomedical machine learning," Nat. Med., vol. 31, no. 4, pp. 1060–1060, Apr. 2025, doi: 10.1038/s41591-025-03637-3.<br>[19]G. Campanella et al., "A clinical benchmark of public self-supervised pathology foundation models," Nat. Commun., vol. 16, no. 1, p. 3640, Apr. 2025, doi: 10.1038/s41467-025-58796-1.<br>[20]A. Zhang, G. Jaume, A. Vaidya, T. Ding, and F. Mahmood, "Accelerating Data Processing and Benchmarking of AI Models for Pathology," Feb. 10, 2025, arXiv: arXiv:2502.06750. doi: 10.48550/arXiv.2502.06750.<br>[21]R. Zahedi et al., "Deep learning in spatially resolved transcriptomics: a comprehensive technical view," Brief. Bioinform., vol. 25, no. 2, p. bbae082, Mar. 2024, doi: 10.1093/bib/bbae082.
**Additional Information:**	
**Question**	**Response**
Are you submitting this manuscript to a special series or article collection?	No

Powered by Editorial Manager® and ProduXion Manager® from Aries Systems Corporation

**Experimental design and statistics**<br><br>Full details of the experimental design and statistical methods used should be given in the Methods section, as detailed in our Minimum Standards Reporting Checklist. Information essential to interpreting the data presented should be made available in the figure legends.<br><br>Have you included all the information requested in your manuscript?	Yes
**Resources**<br><br>A description of all resources used, including antibodies, cell lines, animals and software tools, with enough information to allow them to be uniquely identified, should be included in the Methods section. Authors are strongly encouraged to cite Research Resource Identifiers (RRIDs) for antibodies, model organisms and tools, where possible.<br><br>Have you included the information requested as detailed in our Minimum Standards Reporting Checklist?	Yes
**Availability of data and materials**<br><br>All datasets and code on which the conclusions of the paper rely must be either included in your submission or deposited in publicly available repositories (where available and ethically appropriate), referencing such data using a unique identifier in the references and in the "Availability of Data and Materials" section of your manuscript.<br><br>Have you have met the above requirement as detailed in our Minimum Standards Reporting Checklist?	Yes

Powered by Editorial Manager® and ProduXion Manager® from Aries Systems Corporation

GigaScience has policies and guidelines in place for the use of generative AI-writing tools such as ChatGPT. If you have used such writing tools to assist with writing the manuscript this must be declared and cited in the text. Authors should not list AI-writing tools and other AI-assisted technologies as an author or co-author and should acknowledge that they are fully responsible for text generated or refined by AI-writing tools.<p><br><br>A summary of use (particularly in the introduction or among methods) needs to be included at the end of the paper, and the outputs should also be included as a supplementary file hosted in GigaDB or other open repositories. Please read our guidelines for more information. <p><br><br>By submitting to GigaScience, you are aware of the journal's AI-writing tools policy, and if you have declared use of such tools below, you have acknowledged this where appropriate in your manuscript and have made a summary of use and outputs available. <p><br><b>AI-assisted writing tools have been used in the preparation of this manuscript?	Yes

Manuscript Click here to access/download;Manuscript;manuscript-edits-v2.docx

Emerging AI Approaches for Cancer Spatial Omics

Javad Noorbakhsh[1], Ali Foroughi pour[2], Jeffrey Chuang[1,3]

[1] The Jackson Laboratory for Genomic Medicine, Farmington, CT

[1] St Jude Children’ s Hospital, Memphis, TN

[1] UCONN Health, Department of Genetics and Genome Sciences, Farmington, CT

ORCID:

Javad Noorbakhsh: 0000-0002-6196-8061

Ali Foroughi pour: 0000-0002-3547-0796

Jeffrey Chuang: 0000-0002-3298-2358

Abstract

Technological breakthroughs in spatial omics and artificial intelligence (AI) have the potential to transform the understanding of cancer cells and the tumor microenvironment. Here we review the role of AI in spatial omics, discussing the current state-of-the-art and further needs to decipher cancer biology from large-scale spatial tissue data. An overarching challenge is the development of interpretable spatial AI models, an activity which demands not only improved data integration, but also new conceptual frameworks. We discuss

emerging paradigms – in particular data-driven spatial AI, constraint-based spatial AI, and mechanistic spatial modeling -- as well as the importance of integrating AI with hypothesis-driven strategies and model systems to realize the value of cancer spatial information.

Keywords: artificial intelligence, spatial transcriptomics, spatial proteomics, deep learning, foundation models, tissue biophysics

Background

Recent advances in highly multiplex spatially resolved omics (SRO), such as spatial transcriptomics and proteomics, have led to an explosion of studies on tissue spatial structure and its cellular underpinnings [1], [2]. Such approaches have the potential to revolutionize the histopathologic and molecular understanding of cancer. However, spatial data demand new analysis methods and concepts to address growing challenges in interpretability and reproducibility. Such challenges arise from the vastness of the data space spanning the intricate, yet largely undefined, spatial phenotypes within diverse tissue samples [3], [4]. Novel paradigms for biological discovery are needed to realize the translational value of these rich spatial resources.

SRO data are growing rapidly through imaging technologies, including stain-based (e.g. H&E, IHC), molecular mass-spectrometry (e.g. MALDI),

transcriptomic (e.g. Visium, VisiumHD, Xenium, CosMx), and antibody-based proteomic (e.g. CODEX, CellDive) methods [5]. Low-plex stain-based methods have been widely used in clinical settings for decades. Mass-spectrometry methods are versatile for measuring diverse molecular species, though with associated challenges in specificity [6]. Most prominently, spatial transcriptomic and proteomic methods have accelerated in the past few years, providing high-plex, highly specific quantifications of RNAs and proteins at resolutions of ~0.25-100 microns [7]. These spatial approaches extend single cell and bulk gene-profiling technologies that have been used widely in the last decade [5].

Due to the diversity and complexity of spatial phenomena within tissues, data-driven artificial intelligence approaches, in addition to mechanistic models, will be valuable to obtain insights from SRO data. Large-scale datasets are critical for AI-based data mining, and the scientific community will rely on spatial data being organized in computationally efficient, reusable, and standardized ways. Analogous organizational pressures arose during the high-throughput sequencing revolution, with cancer consortium projects (e.g. TCGA , PCAWG) driving demand for standardization in datatypes (FASTQ, binary alignment: BAM) and genomic annotations (genes, variants, expression), centered around the goal of discovering driver mutations or expression states [8], [9]. More recent consortia (Human Cell Atlas [10], HuBMAP [11], HTAN

[12], SenNet [13], et al) and data aggregation initiatives (e.g CROST [14] and STOmicsDB [15]) have been important for spatial omics data. However, the core goals of tissue spatial analysis have not yet been agreed upon. Despite the community's extensive experience with bulk and dissociated sequencing and protein data, effective goal setting will require openness to new conceptual paradigms.

The most prevalent paradigm for spatial omics analysis has been to extend approaches from single cell analysis, i.e. first, to aggregate sequence (or protein) data for each cell, and second, to analyze how expression relates to cell location [16], [17]; however, this paradigm has inherent limitations. As an illustration, pathologists often make clinically valuable decisions from histology images, which contain no sequence data. Some cancer pathology evaluations may not even depend on cells, for example relying on the morphology and density of blood vessels, the extracellular matrix, or necrotic cavities. Thus, cell-based approaches contain only part of the information valuable in tissue images. To determine the tissue features most relevant to patient outcomes, it will be vital to study not only the value of different data types, but also how to best encode SRO data into useful data representations. The choice of data representation (e.g. cell-based [18], graph-based [19] [20], tile-based [21]) limits the biophysical processes that can be studied, but such limitations have so far been little investigated.

In the following sections we discuss key conceptual challenges and possibilities for the understanding of spatial data, including the role that AI can play. While such topics pertain broadly to tissue biology, spatial profiling has special translational value for cancer. Spatial relationships are critical to treatment response, for example by mediating the interactions of immune and cancer cells during immunotherapy. We conclude by discussing the need for tissue studies within perturbable model systems, which are needed to verify mechanistic understanding and pave the way for clinical translation.

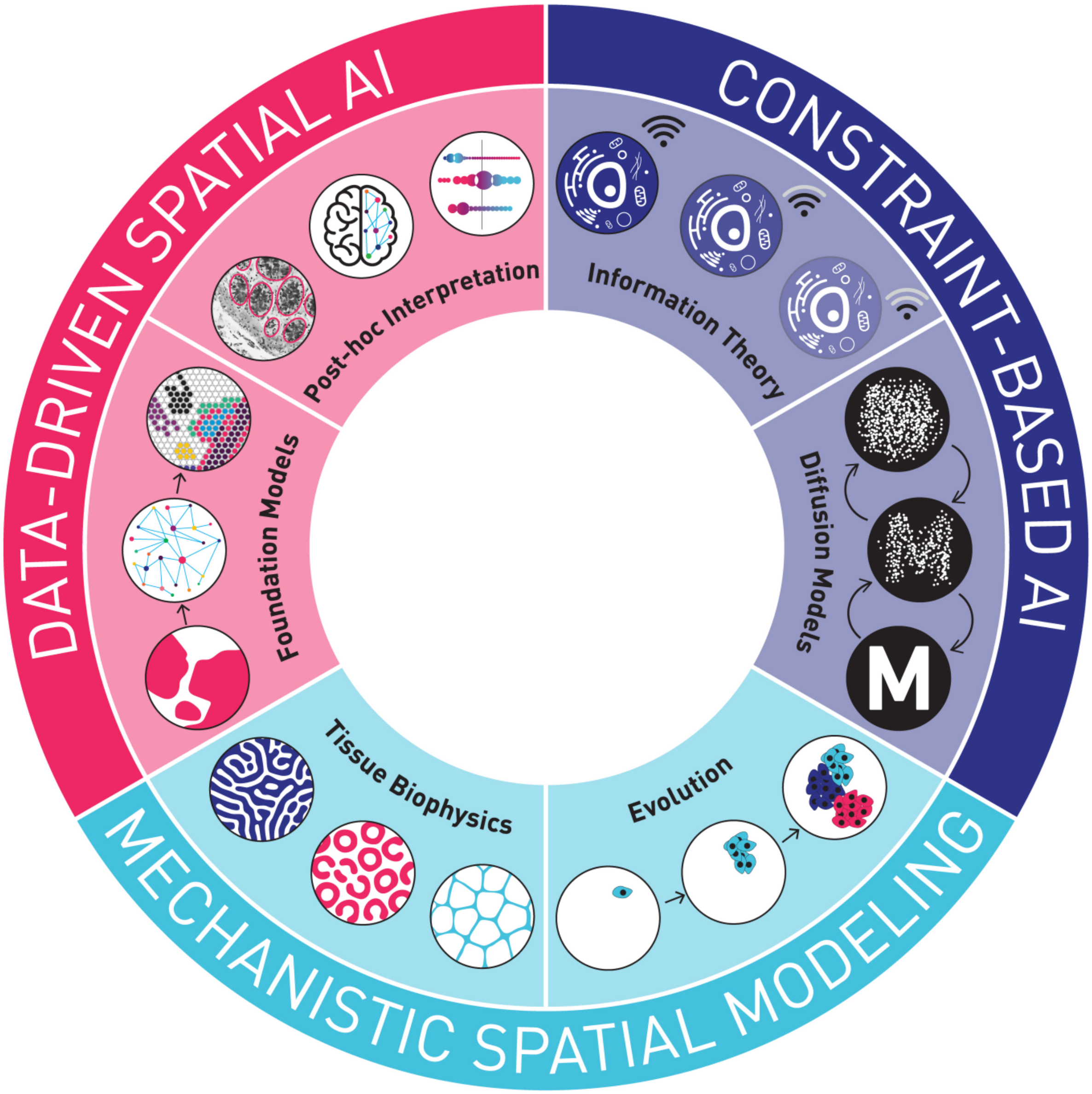

Figure 1. Emerging analysis paradigms for cancer spatial AI. ***DATA-DRIVEN SPATIAL AI*** *is a paradigm that avoids strong assumptions about the underlying data. For example,* ***Foundation Models*** *can be trained on large-scale histopathology or spatial omic data to complete diverse tasks at the level of histopathology, cellular networks, or individual cells. Foundation models typically involve deep neural networks with abstract feature spaces, so* ***Post-hoc Interpretation*** *is needed to identify the structures in the tissue predictive of cancer outcomes. A second paradigm is* ***CONSTRAINT-BASED SPATIAL AI,*** *in which*

intuitive concepts are used to constrain model inference. ***Information Theory*** *is an important framework to guide such models, as cellular interactions in cancer are constrained by information transfer.* ***Diffusion Models*** *are constraint-based AI models used widely in generative image AI and increasingly in tissue analysis, based on the presence of spatial hierarchies in images.* ***MECHANISTIC SPATIAL MODELING*** *is a paradigm based on hypothesis-testing and mechanistic discovery.* ***Tissue Biophysics*** *approaches integrate spatial biophysical concepts (e.g. reaction diffusion, tissue mechanics) with empirical spatial omics observations.* ***Evolution*** *is fundamental to the development and treatment response of tumors, and new spatial omics-based approaches are improving the quantification and understanding of this process.*

Main text

Emerging analysis paradigms for cancer spatial AI

The field of spatial omics is in the early stages of establishing a hierarchy of goals, a prerequisite for the evaluation of data collection and analysis approaches. Nevertheless, several concepts have begun to emerge. Here we review approaches of growing importance, which we organize into three paradigms: Data-driven Spatial AI, Constraint-based Spatial AI, and Mechanistic Spatial Modeling (Figure 1).

Data-driven spatial AI

Many recent spatial analysis algorithms have been data-driven, i.e. they seek to identify patterns in data without pre-specifying biological hypotheses. Such approaches have the advantage of flexibility but can be difficult to interpret mechanistically, particularly when they use deep neural networks to embed biological data into abstract latent spaces. We consider three topics among the data-driven approaches: histopathology foundation models, spatial omic foundation models, and post-hoc interpretation.

Histopathology foundation models

Histopathology foundation models have become a leading category of spatial machine learning models [22], typically involving deep neural network transformer architectures trained on whole slide hematoxylin and eosin (H&E) stained images (Figure 1, Foundation Models). Foundation models are large machine learning models trained on vast datasets, enabling them to tackle diverse tasks [23]. For example, a single foundation model may enable image classification, segmentation, and annotation. In digital and computational pathology, these models have been trained on large H&E slide datasets, in some cases more than one million whole slide images and have demonstrated value for integration into diagnostic workflows [24] [25]. Such foundation models encode

images into data representations which can be used for disease classification, cell segmentation, and outcome prediction. Other clinically-driven image modalities, such as immunohistochemistry (IHC), have also been studied using foundation models [26], but H&E models are the most well-developed, due to the prevalent use of H&E by pathologists for clinical decisions.

Histopathology foundation models are based on deep neural network architectures applied to tiles within H&E whole slide images. Slide representations are then computed by aggregating the behaviors of the tiles. Early foundation models were based on deep convolutional networks [27], but most recent models utilize self-supervised training with a vision transformer [28] backbone. One popular implementation is DINOv2 [29], which seeks to distinguish visual features without model fine-tuning. This architecture has been used in recent foundation models including Virchow [25], UNI [30], and GPFM [31]. Histopathology foundation models have become progressively easier to use, for example having been integrated into end-to-end workflows such as STAMP [32], which facilitates the preprocessing of whole slide H&E images, model training, and evaluation in a single framework.

The wide success of large language models (LLMs), such as GPT [33], BERT [34], and LLaMA [35], has also sparked approaches to merge these with imaging tasks. A recent category of foundation models uses paired H&E images and text (e.g.

CONCH [36], PathChat [37], and TITAN [38]). These approaches combine vision transformers and LLMs to generate integrated data representations and enable chat-based interrogation of histology images.

Spatial omic foundation models

Spatial omics data analysis has benefited from neural network approaches for many tasks, e.g. cell type annotation, batch correction, resolution enhancement, clustering, spatially variable gene detection, dropout imputation, and ligand-receptor detection [39], but general spatial omic foundation models are only beginning to be developed (Figure 1, Foundation Models). It is instructive to compare to dissociated single-cell-based foundation models, which are simpler yet still growing rapidly. For example, a wave of tools leveraging transformers has emerged for single cell deep learning [40]. A key innovation has been in the tokenization step, which specifies what aspects of the input data are important, e.g. gene identity, expression value, ranking, and metadata [41]. For instance, in scGPT [42] tokens comprise genes, expression quantile orders, and experimental conditions. scBERT [43] uses gene2vec [44] to embed co-expression patterns into gene tokens, while binning the expression values. scGPT, scFoundation [45], and CellPLM [46] are foundation models whose resultant embeddings,

whether fine-tuned or direct, can be used as inputs for various tasks. Other models include tGPT [47], xTrimoGene [48], TOSICA [49], and Geneformer [50], and others [40]. A caveat of these foundation models is that the available pretraining datasets have been much smaller than for H&Es and LLMs [40], so caution in accepting their outputs is warranted. Such models should improve as the field matures through improved data, curation, and architectures.

Techniques devised for scRNA foundation models are beginning to be extended to spatial models. Such models also typically rely on transformer architectures trained on large datasets using self-supervision. A few of these models have been introduced as foundation models and can enable multiple downstream tasks. In particular, CellPLM [46] is a transformer encoder/decoder VAE which can do cell clustering, denoising, imputation, and cell type annotation, while Nicheformer [51] is a transformer model capable of label transfer and prediction of cellular neighborhood compositions. Both methods use a combination of spatial and dissociated single cell RNA sequencing (RNAseq) as input, which potentially boosts their ability to transfer information across the two data modalities. Other models such as SpaFormer [52], and stEnTrans [53] are transformer models trained on spatial transcriptomics data which could potentially be utilized as foundational.

Foundation models for spatial proteomics are also a growing area. Spatial proteomic technologies have facilitated in-depth investigation of the tumor microenvironment (TME), offering insights into tumor dynamics and its interactions with the immune system [54] [55]. Advances in multiplex techniques, such as CODEX [56] [57], IMC [58], and Cell Dive [59], have allowed simultaneous measurement of many proteins at subcellular resolution. Such data can enable direct modeling of biophysical processes, including fine interactions such as synapse formation between cells [60]. However, integrating protein data across experiments remains challenging due to variations in protein panels and measured antibody intensity distributions. These issues may be alleviated by encoding protein data into more robust representations, e.g. by non-negative matrix factorization [61] or generative neural networks, which have also enabled powerful capabilities such as combinatorial protein signal decomposition [62] [63]. A promising recent method named KRONOS [64] addresses the issue of protein marker heterogeneity by an innovative tokenization approach. The authors pass the marker identity information as a secondary positional encoding and effectively treat all markers as equivalent otherwise. Their model outperformed existing foundation models that are trained on out-of-domain histology data or multichannel cell profiling images. Still, such encodings may create non-intuitive statistical

artifacts, and broader research into proteomic foundation model development and their benchmarking are important to the further development of the field.

Post-hoc interpretation

Despite their impressive capabilities, foundation models are often criticized as 'black box' systems because their causal logic is not directly interpretable [65] [66]. The loss of interpretability arises from the embedding of image data into abstract latent spaces using deep neural networks. Interpretation of the genetic and cellular features associated with foundation model image embeddings therefore must be *post hoc,* i.e. based on data patterns rather than causal relationships among biological entities (Figure 1, Post-hoc Interpretation). This is in contrast with mechanistic models, which are formulated from experimentally controllable features such as genes or cells.

Some architectures, such as transformer models, provide their *post hoc* interpretability through the attention mechanism, which highlights what input features are focused on by individual attention-heads within the neural network. However, inter-head correlations are difficult to interpret, and attention weights do not uniquely translate to output feature importances [67] [68] [69]. Explainable AI (XAI) techniques like SHAP [70] and LIME [71] address some of these limitations by attributing model predictions to

individual features. SHAP uses a game-theoretic strategy treating features as cooperative players contributing to the prediction, while LIME builds a simpler explainable model through local perturbations of the original model around a data point of interest. In addition to model perturbation, data perturbation can be used to identify biologically plausible perturbations through context-guided data generation [72]. Additionally, methods inspired by physical entropy and thermodynamics have been proposed to identify optimal explanations for model embeddings [73]. These techniques can help capture spatial structures in images that are most indicative of outcomes. Other efforts have been made to develop interpretable models of features related to tissue organization. A useful concept is the functional tissue unit (FTU) [74], defined as the smallest multicellular tissue unit performing a specific function within its microenvironment that is replicated in a whole organ [75]. Repetitive FTUs can be captured by spatial frequency analysis of SRO data. For example, SpaGFT converts the SRO data into a graph and applies a Graph Fourier Transform to identify such features. This method can be implemented as an explainable regularizer for other machine learning models, improving their interpretability [76]. Such modular approaches based on FTUs, even if defined post hoc, are likely to be increasingly used in SRO analysis to demonstrate robustness and reusability of computational frameworks.

However, as technologies for proteomic plexity and throughput improve, deep learning and interpretability considerations will grow for these data types as well [63].

A limitation of post-hoc interpretation approaches is that they reveal associations rather than causal relationships, making them more suited for hypothesis generation. These approaches can inadvertently attribute importance to spurious confounders and batch effects, so their use should be accompanied by batch correction, and ideally, validation on external datasets.

Constraint-based spatial AI

Alternative paradigms based on constraints on the SRO data representation can provide more interpretability than foundation models. For example, all tissue structures are constrained by biophysical processes, but the underlying cellular mechanisms are variable and complex. AI approaches that account for these constraints abstractly, without focusing on individual mechanisms, have grown. We discuss such approaches for cancer, focusing on those incorporating information theoretic constraints and those that mimic spatial constraints, notably image diffusion models.

Information theory-based constraints

Information theory is a mathematical framework for quantifying the flow, processing, and storage of information within systems [77], and it can be applied to guide spatial analysis in tumors. For example, information theoretic concepts can be used to quantify limits on tissue heterogeneity, signaling dynamics, and other types of spatial organization, such as intratumoral immune infiltration and epithelial-to-mesenchymal transitions. Such approaches are helpful for understanding the bounds on inference for different models (Figure 1, Information Theory).

Spatial information transfer in tissues has fundamental limits. This has been demonstrated for drosophila embryos, where morphogen gradients have been optimized by evolution to the physical limits of signal transduction [78]. Such evolutionary optimization likely extends to complex tissues, where information transfer is governed by physical and biological constraints that have been shaped by developmental processes. Viewing this as an information theory problem, with channel capacity and mutual information defining these constraints, provides a powerful analytical framework. In cancer, deviations from these optimal solutions may result in identifiable patterns in SRO data, revealing novel mechanisms driving cytokine, endocrine, and immune signaling [79] [80]. Quantifying signal propagation and interaction provides a way to

understand collective cellular behaviors within cancer tissues driven by external or internal cues.

Spatial constraints on information transfer can motivate SRO dimensionality reduction approaches. For example, non-negative matrix factorization (NMF) has been used to reduce glioma expression data into interpretable patterns of spatially co-expressed genes, i.e. "metaprograms," [81], though it remains challenging to know what spatial scales are appropriate for such approaches. Alternatively, the information bottleneck (IB) method [82] offers an information-theoretic approach to attain the most compressed, lower-dimensional representation of a high-dimensional dataset that is maximally predictive of a desired outcome. bioIB [83] has developed this concept for scRNAseq data to determine a set of genes ('meta-genes') which are predictive of disease status or cell type. Extending such approaches to the spatial domain offers new opportunities for developing more robust SRO data analysis tools.

Extensions of IB have been applied to deep learning models, where the layers in the model are treated as an information channel, and IB is applied to each layer [84] [85]. These layers progressively represent a desired outcome, with each layer 'forgetting' certain input details to better learn the output. Such methods could be extended to spatial data through architectural designs

such as graph neural networks (GNN), convolutional neural networks (CNN), or Vision Transformers (ViT) [28]. This type of strategy may elucidate how tissue-level information flows through molecular and spatial interactions, enabling the extraction of minimal yet predictive feature sets at multiple spatial scales. These minimal encodings could identify molecular signals and spatial arrangements for intercellular communication, revealing key length scales and structural patterns that govern tissue organization and behavior.

Image diffusion models

Diffusion models [86] [87] guided by theoretical frameworks that incorporate tissue-specific symmetries (e.g., repetitive structures, rotational and translational invariance) are a promising approach for spatial analysis (Figure 1, Diffusion Models). These models have achieved significant advances in generative image AI, including widely used tools like DALL-E [88] and Stable Diffusion [89]. Diffusion models are comprised of an encoder that iteratively degrades an image's data distribution structure through a diffusion-like process, followed by a decoder that reverses the process to reconstruct the image with progressive detail. Training such an encoder-decoder on image datasets yields a neural network able to generate images from noise [90]. Latent diffusion models [91] extend traditional diffusion

approaches by first reducing the dimensionality of input data before applying the diffusion process to the compressed representation. This strategy enhances computational efficiency and has been integrated with transformers, forming diffusion transformers (DiTs) [92].

The large number of markers in spatial omics poses a challenge for diffusion models. Embedding data into a lower dimensional space reduces noise, leverages marker correlations, and makes high-dimensional data more manageable. This latent diffusion model approach has been successfully applied to spatial transcriptomics data in methods such as stDiff [93] and SpaDiT [94] which utilize DiTs to impute missing genes, and may be useful for other high dimensional omics types such as mass spectrometry as well. However, a key limitation of these models is their lack of spatial coordinate integration during training, which restricts their ability to fully capture and utilize spatial relationships. Some diffusion models integrate spatial information to better represent the unique characteristics of spatially resolved data. For example, DiffuST [95] employs latent diffusion models alongside graph autoencoders to resolve semantic inconsistencies across data modalities while capturing spatial relationships. SpatialDiffusion [96] incorporates spatial coordinates, gene expression, and cell type information to predict unseen slices in 3D spatial transcriptomics, interpolating distributions from

neighboring slices. Similarly, stMCDI [97] utilizes a graph neural network (GNN) to encode spatial information, leveraging a diffusion model to impute missing data in spatial transcriptomics.

The success of diffusion models is rooted in statistical thermodynamics, as they leverage concepts such as phase transitions, symmetry breaking, and critical instabilities to achieve accurate image reconstruction [98], offering a theoretically-supported approach for generating interpretable representations. While such models have been studied for images with few data channels, extending these principles to high-dimensional spatial omics presents a key challenge important for improving SRO data interpretation [99]. Furthermore, the iterative encoding process in diffusion models parallels the spatial hierarchies within images [100]. Investigation of this relationship may be valuable for improving interpretability. Another promising approach is context-constrained diffusion models [72], which can improve generation of biologically realistic data to facilitate interpretation of foundation models as well as provide augmented data during training.

Mechanistic spatial modeling

Tissue spatial profiling data are, essentially, measurements of 3D materials. In materials science, macroscopic behaviors are studied as mechanistically deriving from microscopic processes such as electromagnetic and molecular

interactions. Likewise, tumors are impacted by microscopic biophysical processes such as chemical signaling and diffusion, resulting in contiguous region types (tumor, immune-infiltrated, necrotic, fibrotic, etc.) with internally coherent cell composition, cellular states, or extracellular structures. Some analysis approaches, e.g. GASTON [101], leverage this coherence to identify region types. However, most current approaches are empirical rather than grounded in biophysical processes. Integration of physics-inspired concepts is therefore a promising direction, and there is a critical need for new AI models capable of mechanistic inference from SRO data. Below we discuss approaches using spatial data to learn biophysical and evolutionary processes within tumors.

Inference of tissue biophysics

Cellular dynamics within tissues can be viewed as a reaction-diffusion process (Figure 1, Tissue Biophysics), where cell-intrinsic mechanisms and cell-to-cell communication occur simultaneously with diffusion. Computational models of cancer based on this perspective have been developed [102] [103] [104], but have been limited in the number of cell types covered and have relied on partial differential equations with many unknown parameters. However, for a hypothesized model of tissue architecture, the governing equations should be

learnable if sufficient data are available. A recent category of inference method for learning equations from large datasets is physics-informed neural networks (PINNs) [105], which embed physical equations into neural networks. These methods require dynamical data for training, and they have been applied to scRNA data to predict cell state dynamics [106] and for RNA velocity inference [107]. So far, application of PINNs to tissues has been limited due to scarcity of time-course data. However, as longitudinal SRO data improve, PINNs can provide a framework to infer interpretable physical processes and parameters from them. Nevertheless, PINNs can still be valuably applied to static data for diffusive systems at steady-state or quasi-steady state, where timescales are sufficiently separated. Methods developed with this philosophy already exist and may be adaptable to PINNs. For example, HoloNet [108] is a graph neural network that uses ligand diffusion equations to infer ligand-receptor interactions from spatial transcriptomics data, and SpaCCC [109] integrates this into a transformer framework. These two methods overcome the limitations of time-course scarcity by stripping chemical diffusion and reaction equations from their dynamics and treating them as steady-state functional forms.

Drawing inspiration from these approaches, it may be possible to model cancer tissue as governed by multiple fields mediated by the spatial diffusion of molecules. An SRO PINN model could be built around cells acting as relays of

such fields—each with its own transmission length scale, which could be inferred from gene or protein expression. These fields could describe, for example, molecular densities or mechanical forces, and cross-attention between them could reveal their interactions, such as ligand-receptor activation. Knowledge of chemical diffusion may be directly incorporated into PINN architecture. For example, multilayer perceptron modules could be used to link gene expression to signal concentration or diffusion rate, while convolution-like kernels could approximate the diffusion process across different length scales. This approach could provide a framework to quantify the spatial dynamics of immune, stromal, and cancer cell populations, as well as their modulation by host factors.

While most neural networks rely on multilayer perceptrons (MLP), which are feed-forward networks with learnable linear edges and fixed nonlinear nodes, Kolmogorov-Arnold Networks (KANs) [110] take the opposite approach, learning nonlinear functions on edges while keeping nodes linear. This design allows KANs to directly infer sub-functional components of a global function, making them potentially more interpretable. Also, although similar to PINNs in their ability to infer system dynamics, KANs are not limited to dynamic data. KANs integrated with convolutional neural networks have been successfully used in remote sensing applications [111] involving geographic spatial profiles at hundreds of light frequencies. Such data are analogous to spatial omics data

in their high number of channels and environment-influenced spatial relationships. Thus, KANs may be effective for interpreting how groups of biomarkers and cell types form structural phenotypes within tissues, using the mathematical functions inferred along KAN edges.

Physical processes such as diffusion can also be qualitatively incorporated into neural networks. For instance, sepal [112] is a spatial transcriptomics analysis tool which assumes that transcripts diffuse in the environment according to a fixed diffusion rate. It then ranks the genes by the time required to reach homogeneity. Although in this method the diffusion process is not physically observed, this approach is nevertheless able to determine spatial structures and their related gene families.

Tissue mechanical properties such as stiffness, adhesion, and viscosity are also important to cancer. These impact tumor invasiveness [113] [114] [115], drug diffusion, and angiogenesis, likely mediated by the impact of mechanical properties on cellular polarity, membrane rigidity, and cell migration [116] [117]. SRO measurements have the potential to reveal mechanical parameters. For example, spatial transcriptomics data has been used to infer tissue stiffness by solving the equations of surface tension from segmented cell membranes [118]. Although the use of cancer SRO data for tissue mechanics studies has been uncommon, the substantial literature on cancer tissue

biophysics [119] [120] suggests that such approaches have untapped potential [121].

Evolution

Evolution has been extensively used to interpret tumor formation, heterogeneity, treatment response, and resistance (Figure 1, Evolution). Many studies have incorporated scRNAseq data toward understanding cancer evolution [122], and analogous spatial transcriptomics-based analysis are growing [123]. Because evolution is classically defined by genotypes, a common use of SRO data has been to optimize spatial genotype calling. Methods like InferCNV [124], which was developed to infer copy number variations (CNV) from scRNAseq data, are now regularly applied to spatial transcriptomics data [125] to identify local CNV-defined genotypes. Analogously, transformer-based CNV-calling methods like CoT [126], which has been used for genome-wide denoising of single cell DNA sequencing (scDNAseq), could be adapted for spatial DNA sequencing. Integrating spatial continuity into these models can improve subclonal phylogeography inference. CalicoST [127] does this, including inference of allele-specific copy numbers, and it has been used to identify subclonal heterogeneity as well as oncogenic and metabolic activity in HTAN datasets [123] . Currently, CalicoST does not leverage long-range spatial

correlations, focusing on spot neighborhoods. However, long-range correlations could arise in images from geographic selection pressures within the tumor, or due to the limited ability of 2D images to represent 3D spatial processes. Improved attention-based networks will be valuable to better capture these effects. PINNs have also been applied to capture tumor growth dynamics [128]. Combining these methods with genomic-based methods may improve phylogeography inference from SRO data.

A broader consideration for tumor evolution is that epigenetic and morphological changes occur together with genotype evolution. For example, epigenetic shifts in tumor cells are important to treatment resistance [125]. Therefore, simultaneous integration of spatial gene expression and phylogenetics in a single network would be valuable. This could be achieved by feeding expression and genotype matrices into a unified tokenization scheme. Tissue morphology, as characterized by H&E and analyzed via foundation models, has also been shown to predict some tumor expression states and genotypes [129] [130]. This suggests that it may be possible to improve inference of tumor evolutionary processes by embedding local genomic data together with the tissue morphology and transcriptional gradient context.

Data considerations

Data integration

Cancer multimodal spatial data are diverse [123], creating data integration opportunities and challenges. At one end, H&E data are widely available but low plex. On the other end, spatial omics datasets are scarce but can have thousands of markers. It remains poorly understood what information is common across modalities and what is modality-specific. To benefit from spatial data integration, technical considerations must be addressed. Different modalities require distinct scaling, standardization, and filtering to avoid biasing downstream models. Image registration across modalities is challenging due to differences in data formats, mismatched markers, resolutions, sample preparation, tissue size, and batch effects [131]. Measurement noise, including technical and biological variability, can complicate data interpretation [132]. Spatial RNA can be affected by platform-specific dropout and probeset limitations [133].

Nevertheless, spatial data integration is already benefiting cancer research. For example, cancer H&E images have been shown to predict spatial expression-identified patterns including immune infiltration and drug-induced persistence [125], [21], [134]. Pipelines are available to ease H&E/spatial transcriptomic

integration for cancer, such as STQ [135]. Other general integration methods will also be useful for cancer. For example, SpatialGlue [136] integrates spatial transcriptomics, proteomics and epigenomics data for domain identification. Architectures based on single-cell integration frameworks such as scGPT [42] and scMoFormer [137] provide a model for expanding spatial data integration. COVET [138] uses localized expression covariances to encode cellular niches in spatial transcriptomics data. ENVI [138] is a variational autoencoder that integrates with COVET to simultaneously infer missing genes in spatial transcriptomics data inferred from dissociated single cell data, while assigning niche information to dissociated cells using spatial data. Approaches based on large-scale H&E integration with expression data are also of growing value [139]. For example, OmiCLIP [140] encodes highly expressed genes from local spots into sentences, then uses vision-language deep learning theory to build an H&E-omics foundation model.

Different spatial omics modalities from the same tissue often have mismatched resolution, complicating integration. Heterogenous GNNs [141], which assign modality-specific attributes to nodes, have been used to study the tumor microenvironment in spatial transcriptomics data [142] and could potentially support multi-resolution integration by linking nodes (e.g. cells or spots) by spatial proximity. Similarly, multi-resolution transformers have shown success in histopathology [143] by jointly processing images at multiple zoom scales.

A similar strategy could be adapted for spatial omics integration by treating each resolution as a distinct modality.

Platform-specific noise (e.g. spatial RNA dropout) further complicates integration. Methods adapted from single-cell analysis can help mitigate this issue. For example, LLOKI [144] combines neighborhood and distributional similarity and pretrained single-cell foundation models to reduce expression sparsity and correct batch effects across datasets. Divergent marker panels, particularly in spatial proteomics, also impede integration. This can be mitigated by recognizing that phenotypes often recur even when exact markers differ. Integration with other data types (e.g. H&E) and marker-agnostic encoding methods [64] can be effective as well.

Model benchmarking

Rapid progress in digital pathology foundation models make rigorous benchmarking essential. Current *de facto* H&E benchmarks, such as TCGA subtype classification and molecular prediction [30], [145], largely reflect historical data availability rather than a coordinated community-driven effort aimed at biological discovery and translational impact [146]. To close this gap, the field needs community initiatives to curate agreed-upon public datasets and standardize metrics. While clinically grounded datasets and benchmarks are emerging [147], [148], broad institutional momentum remains

limited; nonetheless, we anticipate increasing standardization in the coming years.

In the SRO domain the need is even more acute, as limited large-scale datasets and rapidly evolving assays make benchmarking ambiguous. Existing benchmarks mostly target unsupervised tasks (e.g. spatial domain identification) or self-supervised objectives (e.g. imputing missing genes), or rely on expert-annotated datasets that are small and domain-specific [39]. Where labels exist, they are typically intra-tissue (e.g. cell type annotations) rather than cross-sample, and patient-level annotation is scarce. These benchmarks are valuable for research, but to build parallels to the H&E AI models would require clinically grounded benchmarks built on large-scale patient-level annotations. Organizing consortia to generate clinically annotated, patient-derived spatial omics datasets is essential to establish relevant ground truth and enable clinically meaningful benchmarking.

Mechanism-driven data generation

Spatial data have tremendous potential for improving prediction of clinical outcomes (e.g. [149]) while simultaneously suggesting new mechanisms. However, post-hoc interpretation approaches conflict with hypothesis-oriented standards for mechanistic discovery. For example, cancer clinical H&E samples have been studied extensively by foundation model approaches, but it remains unclear how

to ascertain causal relationships from them. Patient IRB considerations also limit the types of clinical samples that can be obtained. Such issues will constrain human SRO-based foundation model approaches, even if large cancer datasets can be amassed [150]. To verify causal mechanisms, it will be important to purposefully generate spatial data for mechanistic discovery.

Mice are the canonical mammalian model organism, making them ideal for the study of cancer spatial mechanisms in a tissue context. The ease of mouse genetic engineering and organismal perturbation has a long history of enabling hypothesis-based discoveries of oncogenes, tumor suppressors, metastatic processes, tumor environmental interactions, and effects of aging [151]. Sophisticated mouse population genetic systems such as the Diversity Outbred and Collaborative Cross [152] have also revealed genes important to cancer mechanisms [153] and quantitative trait loci predictive of cell morphology [154]. Dynamic SRO data can be generated more easily in mice than from clinical samples, a key need for the training of PINNs. Stated simply, mice and other organismal models enable hypothesis-driven science on tissues, addressing the central challenge of post hoc mechanistic interpretation.

To realize the value of mouse and other animal model SRO cancer studies, such knowledge must be transferable to human. Identification of orthologous behaviors between mouse and human has not yet been well-quantified with SRO

data, though projects such as the Cellular Senescence Network (SenNet) [13] are performing spatial profiling of tissues across some matched mouse and human organs. Methods for the scRNA version of this problem are being actively developed. For example, CAME [155] aligns scRNAseq data across species and enables the transfer of cell type labels, which it accomplishes via a graph neural network that integrates a gene-gene graph of homologous genes and a cell-cell graph of transcriptionally similar cells. BrainAlign [156] extends this idea to spatial transcriptomics by adding a graph linking spatially proximal spots on the tissue. BrainAlign can align human and mouse brain tissue as well as identify conserved and species-specific gene expression patterns. Existing SRO transformer models may also be developed for human and mouse SRO comparisons. For example, Nicheformer [51] uses a unified gene-based tokenizer for human and mouse spatial transcriptomics and single cell RNAseq, producing embeddings for tasks such as spatial label prediction. However, it does not use spatial coordinates as input. In contrast, SpaFormer [52] uses a cell-based tokenizer and explicitly encodes positional information to impute missing spatial transcriptomics data. A combined architecture with multi-species tokenizers and explicit positional encodings could better align human and mouse SRO data. All these approaches will require further development of expert annotated SRO sets in mouse and human to train cross-species spatial aligners. Despite evolutionary similarities, human and mouse may diverge in

many aspects of tissue architecture including fine immune microenvironmental organization and gene expression dynamics [157]. Computational mapping of tissue architecture across the two species is a broad challenge whose solution would improve the translational use of mouse models for human cancer research.

Conclusions

The field of spatial omics is expanding rapidly in cancer research due to the critical importance of location-dependent interactions of cells within the tumor microenvironment. Cancer spatial omics datasets are high-dimensional and diverse, necessitating improved analytical paradigms. We have described three major paradigms for the development of the field: data-driven spatial AI; constraint-based spatial AI; and mechanistic spatial modeling. We have also reviewed key additional considerations in data integration and mechanism-driven data generation.

While the scale and complexity of SRO data create strong demand for AI-based approaches, deep neural networks have only post-hoc interpretability compared to classical hypothesis-oriented approaches. Among the three major paradigms, deep neural network-based data-driven approaches, such as foundation models, are the least interpretable or mechanistic. Constraint-based spatial AI, such as image diffusion models, improve on this by incorporating biologically

reasonable constraints into the underlying neural networks. Mechanistic models are the most interpretable, as they are constructed based on experimentally perturbable entities such as cells or genes. PINNs are a particularly promising approach for mechanistic modeling, as they combine explicit mathematical modeling of perturbable entities with data-driven inference amenable to SRO data. This provides a framework to jointly study biophysical and cell biological processes within tissues. Such joint investigation is essential to the underlying spatial processes of cell motility, signaling, replication, and evolution within the tumor microenvironment.

A general challenge for spatial omics foundation models is validation. Although tests such as tumor-type classification are commonly accepted for H&E foundation model benchmarking, spatial omics foundation model benchmarking is not yet standardized. To be useful, SRO foundation models should be able to distinguish fine microenvironments within tumors, a more complex task that is also inherently multiscale. Thus, SRO foundation model benchmarking will require community agreement on standardized evaluation tasks at multiple spatial scales. Alternative statistical models may be valuable in circumventing these challenges, e.g. constraint-based statistical models can be more interpretable and require less training data than deep learning counterparts [158]. Models based on message passing are a canonical example, where information of neighboring cells are aggregated to represent the

cellular composition and spatial relations [159]. These models share similarities with GNNs but require less training data. Interestingly, recent studies have combined GNNs with message passing techniques to generate improved embeddings given small data [160] [161] [162].

While spatial data are growing for clinical cancer samples, clinical data are restricted by regulatory and collection limitations. These restrictions make it difficult to generate the perturbative data important for hypothesis-based science or the time-courses important for training of PINNs. Mouse models can address this problem, as biophysical and genetic perturbations are possible with mice, and time-course data are easier to generate with mice than in the clinic. Still, mice may respond differently to treatment than patients, and SRO-based models trained on mice will likely need to be fine-tuned on human data. Eventually, curated repositories that integrate human and mouse SRO cancer tissue data will be vital for the field. These will enable improved identification and validation of functional tissue units, reusable data analysis, and more precise delineation of the spatial processes essential to cancer marker identification, drug targeting, and clinical translation.

List of abbreviations

AI – Artificial Intelligence
BAM – Binary Alignment Map

BERT – Bidirectional Encoder Representations from Transformers
CNN – Convolutional Neural Network
CNV – Copy Number Variation
CODEX – CO-Detection by indEXing
CONCH – Context-aware Chat-based Histopathology
DNAseq – DNA Sequencing
DiT – Diffusion Transformer
FTU – Functional Tissue Unit
GNN – Graph Neural Network
GPT – Generative Pre-trained Transformer
H&E – Hematoxylin and Eosin
HTAN – Human Tumor Atlas Network
IB – Information Bottleneck
IHC – Immunohistochemistry
IMC – Imaging Mass Cytometry
IRB – Institutional Review Board
KAN – Kolmogorov-Arnold Network
LIME – Local Interpretable Model-agnostic Explanations
LLM – Large Language Model
MALDI – Matrix-Assisted Laser Desorption/Ionization
MLP – Multilayer Perceptron
NMF – Non-negative Matrix Factorization
PCAWG – Pan-Cancer Analysis of Whole Genomes
PINN – Physics-Informed Neural Network
RNAseq – RNA Sequencing
SHAP – SHapley Additive exPlanations
SRO – Spatially Resolved Omics
SenNet – The Cellular Senescence Network
TCGA – The Cancer Genome Atlas
TME – Tumor Microenvironment
ViT – Vision Transformer
XAI – Explainable Artificial Intelligence
scDNAseq – Single-cell DNA sequencing
scRNAseq – Single-cell RNA sequencing

Declarations

Data availability: Not applicable

Competing interests: The authors declare no competing interests

Funding: The authors acknowledge support from The Jackson Laboratory Cancer Center's Cancer Advanced Technology (CATch) program, as well as NIH grants R01 CA230031, U54 AG075941, and P30 CA034196

Author's contribution: JN and JC planned and drafted the original version of the manuscript. AFP reviewed, provided new ideas, and drafted revisions.

Acknowledgements: The authors thank Karolina Palucka, Brian White, Frederick Varn, Francesca Menghi, and Kevin Anderson for valuable discussions.

References

[1] D. Bressan, G. Battistoni, and G. J. Hannon, "The dawn of spatial omics," *Science*, vol. 381, no. 6657, p. eabq4964, Aug. 2023, doi: 10.1126/science.abq4964.

[2] J. L. Carstens, S. N. Krishnan, A. Rao, *et al.*, "Spatial multiplexing and omics," *Nat. Rev. Methods Primer*, vol. 4, no. 1, p. 54, Aug. 2024, doi: 10.1038/s43586-024-00330-6.

[3] M. Cheng, Y. Jiang, J. Xu, *et al.*, "Spatially resolved transcriptomics: a comprehensive review of their technological advances, applications, and challenges," *J. Genet. Genomics*, vol. 50, no. 9, pp. 625–640, Sept. 2023, doi: 10.1016/j.jgg.2023.03.011.

[4] S. Fang, B. Chen, Y. Zhang, *et al.*, "Computational Approaches and Challenges in Spatial Transcriptomics," *Genomics Proteomics Bioinformatics*, vol. 21, no. 1, pp. 24–47, Feb. 2023, doi: 10.1016/j.gpb.2022.10.001.

[5] A. Kulasinghe, N. Berrell, M. L. Donovan, *et al.*, "Spatial-Omics Methods and Applications," in *Gene Expression Analysis: Methods and Protocols*, N. Raghavachari and N. Garcia-Reyero, Eds., New York, NY: Springer US, 2025, pp. 101–146. doi: 10.1007/978-1-0716-4276-4_5.

[6] H. Zhang, K. H. Lu, M. Ebbini, *et al.*, "Mass spectrometry imaging for spatially resolved multi-omics molecular mapping," *Npj Imaging*, vol. 2, no. 1, p. 20, July 2024, doi: 10.1038/s44303-024-00025-3.

[7] L. Liu, A. Chen, Y. Li, *et al.*, "Spatiotemporal omics for biology and medicine," *Cell*, vol. 187, no. 17, pp. 4488–4519, Aug. 2024, doi: 10.1016/j.cell.2024.07.040.
[8] M. J. Goldman, J. Zhang, N. A. Fonseca, *et al.*, "A user guide for the online exploration and visualization of PCAWG data," *Nat. Commun.*, vol. 11, no. 1, p. 3400, July 2020, doi: 10.1038/s41467-020-16785-6.
[9] Z. Zhang, K. Hernandez, J. Savage, *et al.*, "Uniform genomic data analysis in the NCI Genomic Data Commons," *Nat. Commun.*, vol. 12, no. 1, p. 1226, Feb. 2021, doi: 10.1038/s41467-021-21254-9.
[10] A. Regev, S. A. Teichmann, E. S. Lander, *et al.*, "The Human Cell Atlas," *eLife*, vol. 6, p. e27041, Dec. 2017, doi: 10.7554/eLife.27041.
[11] M. P. Snyder, S. Lin, A. Posgai, *et al.*, "The human body at cellular resolution: the NIH Human Biomolecular Atlas Program," *Nature*, vol. 574, no. 7777, pp. 187–192, Oct. 2019, doi: 10.1038/s41586-019-1629-x.
[12] "Tumour atlases enable researchers to navigate through cancers," *Nature*, Oct. 2024, doi: 10.1038/d41586-024-03498-9.
[13] A. U. Gurkar, A. A. Gerencser, A. L. Mora, *et al.*, "Spatial mapping of cellular senescence: emerging challenges and opportunities," *Nat. Aging*, vol. 3, no. 7, pp. 776–790, July 2023, doi: 10.1038/s43587-023-00446-6.
[14] G. Wang, S. Wu, Z. Xiong, *et al.*, "CROST: a comprehensive repository of spatial transcriptomics," *Nucleic Acids Res.*, vol. 52, no. D1, pp. D882–D890, Jan. 2024, doi: 10.1093/nar/gkad782.
[15] Z. Xu, W. Wang, T. Yang, *et al.*, "STOmicsDB: a comprehensive database for spatial transcriptomics data sharing, analysis and visualization," *Nucleic Acids Res.*, vol. 52, no. D1, pp. D1053–D1061, Jan. 2024, doi: 10.1093/nar/gkad933.
[16] J. Du, Y.-C. Yang, Z.-J. An, *et al.*, "Advances in spatial transcriptomics and related data analysis strategies," *J. Transl. Med.*, vol. 21, no. 1, p. 330, May 2023, doi: 10.1186/s12967-023-04150-2.
[17] R. Dries, J. Chen, N. del Rossi, *et al.*, "Advances in spatial transcriptomic data analysis," *Genome Res.*, vol. 31, no. 10, pp. 1706–1718, Oct. 2021, doi: 10.1101/gr.275224.121.
[18] G. Palla, H. Spitzer, M. Klein, *et al.*, "Squidpy: a scalable framework for spatial omics analysis," *Nat. Methods*, vol. 19, no. 2, pp. 171–178, Feb. 2022, doi: 10.1038/s41592-021-01358-2.
[19] J. Hu, X. Li, K. Coleman, *et al.*, "SpaGCN: Integrating gene expression, spatial location and histology to identify spatial domains and spatially variable genes by graph convolutional network," *Nat. Methods*, vol. 18, no. 11, pp. 1342–1351, Nov. 2021, doi: 10.1038/s41592-021-01255-8.

[20] T. Liu, Z.-Y. Fang, Z. Zhang, *et al.*, "A comprehensive overview of graph neural network-based approaches to clustering for spatial transcriptomics," *Comput. Struct. Biotechnol. J.*, vol. 23, pp. 106–128, Dec. 2024, doi: 10.1016/j.csbj.2023.11.055.
[21] B. He, L. Bergenstråhle, L. Stenbeck, *et al.*, "Integrating spatial gene expression and breast tumour morphology via deep learning," *Nat. Biomed. Eng.*, vol. 4, no. 8, pp. 827–834, Aug. 2020, doi: 10.1038/s41551-020-0578-x.
[22] C. Xiong, H. Chen, and J. J. Y. Sung, "A Survey of Pathology Foundation Model: Progress and Future Directions," May 21, 2025, *arXiv*: arXiv:2504.04045. doi: 10.48550/arXiv.2504.04045.
[23] R. Bommasani, D. A. Hudson, E. Adeli, *et al.*, "On the Opportunities and Risks of Foundation Models," July 12, 2022, *arXiv*: arXiv:2108.07258. doi: 10.48550/arXiv.2108.07258.
[24] D. Nechaev, A. Pchelnikov, and E. Ivanova, "Hibou: A Family of Foundational Vision Transformers for Pathology," Aug. 20, 2024, *arXiv*: arXiv:2406.05074. doi: 10.48550/arXiv.2406.05074.
[25] E. Vorontsov, A. Bozkurt, A. Casson, *et al.*, "A foundation model for clinical-grade computational pathology and rare cancers detection," *Nat. Med.*, vol. 30, no. 10, pp. 2924–2935, Oct. 2024, doi: 10.1038/s41591-024-03141-0.
[26] J. Dippel, B. Feulner, T. Winterhoff, *et al.*, "RudolfV: A Foundation Model by Pathologists for Pathologists," June 11, 2024, *arXiv*: arXiv:2401.04079. doi: 10.48550/arXiv.2401.04079.
[27] M. Springenberg, A. Frommholz, M. Wenzel, *et al.*, "From modern CNNs to vision transformers: Assessing the performance, robustness, and classification strategies of deep learning models in histopathology," *Med. Image Anal.*, vol. 87, p. 102809, July 2023, doi: 10.1016/j.media.2023.102809.
[28] A. Dosovitskiy, L. Beyer, A. Kolesnikov, *et al.*, "An Image is Worth 16x16 Words: Transformers for Image Recognition at Scale," presented at the International Conference on Learning Representations, Oct. 2020. Available: https://openreview.net/forum?id=YicbFdNTTy
[29] M. Oquab, T. Darcet, T. Moutakanni, *et al.*, "DINOv2: Learning Robust Visual Features without Supervision," *Trans. Mach. Learn. Res.*, July 2023, Available: https://openreview.net/forum?id=a68SUt6zFt
[30] R. J. Chen, T. Ding, M. Y. Lu, *et al.*, "Towards a general-purpose foundation model for computational pathology," *Nat. Med.*, vol. 30, no. 3, pp. 850–862, Mar. 2024, doi: 10.1038/s41591-024-02857-3.
[31] J. Ma, Z. Guo, F. Zhou, *et al.*, "A generalizable pathology foundation model using a unified knowledge distillation pretraining framework,"

Nat. Biomed. Eng., pp. 1–20, Sept. 2025, doi: 10.1038/s41551-025-01488-4.

[32] O. S. M. El Nahhas, M. van Treeck, G. Wölflein, *et al.*, "From whole-slide image to biomarker prediction: end-to-end weakly supervised deep learning in computational pathology," *Nat. Protoc.*, pp. 1–24, Sept. 2024, doi: 10.1038/s41596-024-01047-2.

[33] T. Brown, B. Mann, N. Ryder, *et al.*, "Language Models are Few-Shot Learners," in *Advances in Neural Information Processing Systems*, Curran Associates, Inc., 2020, pp. 1877–1901. Available: https://papers.nips.cc/paper/2020/hash/1457c0d6bfcb4967418bfb8ac142f64a-Abstract.html

[34] J. Devlin, M.-W. Chang, K. Lee, *et al.*, "BERT: Pre-training of Deep Bidirectional Transformers for Language Understanding," in *Proceedings of the 2019 Conference of the North American Chapter of the Association for Computational Linguistics: Human Language Technologies, Volume 1 (Long and Short Papers)*, Minneapolis, Minnesota: Association for Computational Linguistics, June 2019, pp. 4171–4186. doi: 10.18653/v1/N19-1423.

[35] H. Touvron, T. Lavril, G. Izacard, *et al.*, "LLaMA: Open and Efficient Foundation Language Models," Feb. 27, 2023, *arXiv*: arXiv:2302.13971. doi: 10.48550/arXiv.2302.13971.

[36] M. Y. Lu, B. Chen, D. F. K. Williamson, *et al.*, "A visual-language foundation model for computational pathology," *Nat. Med.*, vol. 30, no. 3, pp. 863–874, Mar. 2024, doi: 10.1038/s41591-024-02856-4.

[37] M. Y. Lu, B. Chen, D. F. K. Williamson, *et al.*, "A multimodal generative AI copilot for human pathology," *Nature*, vol. 634, no. 8033, pp. 466–473, Oct. 2024, doi: 10.1038/s41586-024-07618-3.

[38] T. Ding, S. J. Wagner, A. H. Song, *et al.*, "Multimodal Whole Slide Foundation Model for Pathology," Nov. 29, 2024, *arXiv*: arXiv:2411.19666. doi: 10.48550/arXiv.2411.19666.

[39] R. Zahedi, R. Ghamsari, A. Argha, *et al.*, "Deep learning in spatially resolved transcriptomics: a comprehensive technical view," *Brief. Bioinform.*, vol. 25, no. 2, p. bbae082, Mar. 2024, doi: 10.1093/bib/bbae082.

[40] A. Sza ata, K. Hrovatin, S. Becker, *et al.*, "Transformers in single-cell omics: a review and new perspectives," *Nat. Methods*, vol. 21, no. 8, pp. 1430–1443, Aug. 2024, doi: 10.1038/s41592-024-02353-z.

[41] Z. Sims, S. Govindarajan, G. Mills, *et al.*, "Language of Stains: Tokenization Enhances Multiplex Immunofluorescence and Histology Image Synthesis," Mar. 11, 2025, *bioRxiv*. doi: 10.1101/2025.03.04.641512.

[42] H. Cui, C. Wang, H. Maan, *et al.*, "scGPT: toward building a foundation model for single-cell multi-omics using generative AI," *Nat. Methods*, vol. 21, no. 8, pp. 1470–1480, Aug. 2024, doi: 10.1038/s41592-024-02201-0.
[43] F. Yang, W. Wang, F. Wang, *et al.*, "scBERT as a large-scale pretrained deep language model for cell type annotation of single-cell RNA-seq data," *Nat. Mach. Intell.*, vol. 4, no. 10, pp. 852–866, Oct. 2022, doi: 10.1038/s42256-022-00534-z.
[44] J. Du, P. Jia, Y. Dai, *et al.*, "Gene2vec: distributed representation of genes based on co-expression," *BMC Genomics*, vol. 20, no. 1, p. 82, Feb. 2019, doi: 10.1186/s12864-018-5370-x.
[45] M. Hao, J. Gong, X. Zeng, *et al.*, "Large-scale foundation model on single-cell transcriptomics," *Nat. Methods*, vol. 21, no. 8, pp. 1481–1491, Aug. 2024, doi: 10.1038/s41592-024-02305-7.
[46] H. Wen, W. Tang, X. Dai, *et al.*, "CellPLM: Pre-training of Cell Language Model Beyond Single Cells," presented at the The Twelfth International Conference on Learning Representations, Oct. 2023. Available: https://openreview.net/forum?id=BKXvPDekud
[47] H. Shen, J. Liu, J. Hu, *et al.*, "Generative pretraining from large-scale transcriptomes for single-cell deciphering," *iScience*, vol. 26, no. 5, p. 106536, May 2023, doi: 10.1016/j.isci.2023.106536.
[48] J. Gong, M. Hao, X. Cheng, *et al.*, "xTrimoGene: An Efficient and Scalable Representation Learner for Single-Cell RNA-Seq Data," presented at the Thirty-seventh Conference on Neural Information Processing Systems, Nov. 2023. Available: https://openreview.net/forum?id=gdwcoBCMVi
[49] J. Chen, H. Xu, W. Tao, *et al.*, "Transformer for one stop interpretable cell type annotation," *Nat. Commun.*, vol. 14, no. 1, p. 223, Jan. 2023, doi: 10.1038/s41467-023-35923-4.
[50] C. V. Theodoris, L. Xiao, A. Chopra, *et al.*, "Transfer learning enables predictions in network biology," *Nature*, vol. 618, no. 7965, pp. 616–624, June 2023, doi: 10.1038/s41586-023-06139-9.
[51] A. C. Schaar, A. Tejada-Lapuerta, G. Palla, *et al.*, "Nicheformer: a foundation model for single-cell and spatial omics," Apr. 17, 2024, *bioRxiv*. doi: 10.1101/2024.04.15.589472.
[52] H. Wen, W. Tang, W. Jin, *et al.*, "Single Cells Are Spatial Tokens: Transformers for Spatial Transcriptomic Data Imputation," Feb. 16, 2024, *arXiv*: arXiv:2302.03038. Available: http://arxiv.org/abs/2302.03038
[53] S. Xue, F. Zhu, C. Wang, *et al.*, "stEnTrans: Transformer-Based Deep Learning for Spatial Transcriptomics Enhancement," in *Bioinformatics*

Research and Applications: 20th International Symposium, ISBRA 2024, Kunming, China, July 19–21, 2024, Proceedings, Part I, Berlin, Heidelberg: Springer-Verlag, July 2024, pp. 63–75. doi: 10.1007/978-981-97-5128-0_6.
[54] J. M. Carter, S. Chumsri, D. A. Hinerfeld, *et al.*, "Distinct spatial immune microlandscapes are independently associated with outcomes in triple-negative breast cancer," *Nat. Commun.*, vol. 14, no. 1, p. 2215, Apr. 2023, doi: 10.1038/s41467-023-37806-0.
[55] S. L. Shiao, K. H. Gouin, N. Ing, *et al.*, "Single-cell and spatial profiling identify three response trajectories to pembrolizumab and radiation therapy in triple negative breast cancer," *Cancer Cell*, vol. 42, no. 1, pp. 70-84.e8, Jan. 2024, doi: 10.1016/j.ccell.2023.12.012.
[56] Y. Goltsev, N. Samusik, J. Kennedy-Darling, *et al.*, "Deep Profiling of Mouse Splenic Architecture with CODEX Multiplexed Imaging," *Cell*, vol. 174, no. 4, pp. 968-981.e15, Aug. 2018, doi: 10.1016/j.cell.2018.07.010.
[57] J. Kennedy-Darling, S. S. Bhate, J. W. Hickey, *et al.*, "Highly multiplexed tissue imaging using repeated oligonucleotide exchange reaction," *Eur. J. Immunol.*, vol. 51, no. 5, pp. 1262–1277, 2021, doi: 10.1002/eji.202048891.
[58] C. Giesen, H. A. O. Wang, D. Schapiro, *et al.*, "Highly multiplexed imaging of tumor tissues with subcellular resolution by mass cytometry," *Nat. Methods*, vol. 11, no. 4, pp. 417–422, Apr. 2014, doi: 10.1038/nmeth.2869.
[59] A. J. Radtke, C. J. Chu, Z. Yaniv, *et al.*, "IBEX: an iterative immunolabeling and chemical bleaching method for high-content imaging of diverse tissues," *Nat. Protoc.*, vol. 17, no. 2, pp. 378–401, Feb. 2022, doi: 10.1038/s41596-021-00644-9.
[60] V. G. Wang, Z. Liu, J. Martinek, *et al.*, "Computational immune synapse analysis reveals T-cell interactions in distinct tumor microenvironments," *Commun. Biol.*, vol. 7, no. 1, pp. 1–17, Sept. 2024, doi: 10.1038/s42003-024-06902-2.
[61] A. F. Pour, T.-C. Wu, J. Martinek, *et al.*, "Prediction of Outcome from Spatial Protein Profiling of Triple-Negative Breast Cancers," Apr. 18, 2025, *bioRxiv*. doi: 10.1101/2025.04.18.649541.
[62] R. Ben-Uri, L. Ben Shabat, D. Shainshein, *et al.*, "High-dimensional imaging using combinatorial channel multiplexing and deep learning," *Nat. Biotechnol.*, pp. 1–14, Mar. 2025, doi: 10.1038/s41587-025-02585-0.
[63] S. Ayub, H. W. Jackson, A. Selega, *et al.*, "Multi-view deep learning of highly multiplexed imaging data improves association of cell states with clinical outcomes," Mar. 17, 2025, *bioRxiv*. doi: 10.1101/2025.03.14.643377.

[64]M. Shaban, Y. Chang, H. Qiu, *et al.*, “A Foundation Model for Spatial Proteomics,” June 05, 2025, *arXiv*: arXiv:2506.03373. doi: 10.48550/arXiv.2506.03373.
[65]C. Rudin, “Stop explaining black box machine learning models for high stakes decisions and use interpretable models instead,” *Nat. Mach. Intell.*, vol. 1, no. 5, pp. 206–215, May 2019, doi: 10.1038/s42256-019-0048-x.
[66]D. Alvarez Melis and T. Jaakkola, “Towards Robust Interpretability with Self-Explaining Neural Networks,” in *Advances in Neural Information Processing Systems*, Curran Associates, Inc., 2018. Available: https://papers.nips.cc/paper_files/paper/2018/hash/3e9f0fc9b2f89e043bc6233994dfcf76-Abstract.html
[67]S. Jain and B. C. Wallace, “Attention is not Explanation,” in *Proceedings of the 2019 Conference of the North American Chapter of the Association for Computational Linguistics: Human Language Technologies, Volume 1 (Long and Short Papers)*, J. Burstein, C. Doran, and T. Solorio, Eds., Minneapolis, Minnesota: Association for Computational Linguistics, June 2019, pp. 3543–3556. doi: 10.18653/v1/N19-1357.
[68]S. Serrano and N. A. Smith, “Is Attention Interpretable?,” in *Proceedings of the 57th Annual Meeting of the Association for Computational Linguistics*, A. Korhonen, D. Traum, and L. Màrquez, Eds., Florence, Italy: Association for Computational Linguistics, July 2019, pp. 2931–2951. doi: 10.18653/v1/P19-1282.
[69]B. Bai, J. Liang, G. Zhang, *et al.*, “Why Attentions May Not Be Interpretable?,” in *Proceedings of the 27th ACM SIGKDD Conference on Knowledge Discovery & Data Mining*, in KDD ’21. New York, NY, USA: Association for Computing Machinery, Aug. 2021, pp. 25–34. doi: 10.1145/3447548.3467307.
[70]S. M. Lundberg and S.-I. Lee, “A unified approach to interpreting model predictions,” in *Proceedings of the 31st International Conference on Neural Information Processing Systems*, in NIPS’17. Red Hook, NY, USA: Curran Associates Inc., Dec. 2017, pp. 4768–4777.
[71]M. T. Ribeiro, S. Singh, and C. Guestrin, “‘Why Should I Trust You?’: Explaining the Predictions of Any Classifier,” in *Proceedings of the 22nd ACM SIGKDD International Conference on Knowledge Discovery and Data Mining*, in KDD ’16. New York, NY, USA: Association for Computing Machinery, Aug. 2016, pp. 1135–1144. doi: 10.1145/2939672.2939778.
[72]K. Islam and N. Akhtar, “Context-Guided Responsible Data Augmentation with Diffusion Models,” presented at the ICLR 2025 Workshop on Navigating and Addressing Data Problems for Foundation Models, Mar. 2025. Available: https://openreview.net/forum?id=N4SmKuXddZ

[73] S. Mehdi and P. Tiwary, “Thermodynamics-inspired explanations of artificial intelligence,” *Nat. Commun.*, vol. 15, no. 1, p. 7859, Sept. 2024, doi: 10.1038/s41467-024-51970-x.
[74] K. Börner, A. Bueckle, B. W. Herr, *et al.*, “Tissue registration and exploration user interfaces in support of a human reference atlas,” *Commun. Biol.*, vol. 5, no. 1, pp. 1–9, Dec. 2022, doi: 10.1038/s42003-022-03644-x.
[75] S. Jain, L. Pei, J. M. Spraggins, *et al.*, “Advances and prospects for the Human BioMolecular Atlas Program (HuBMAP),” *Nat. Cell Biol.*, vol. 25, no. 8, pp. 1089–1100, Aug. 2023, doi: 10.1038/s41556-023-01194-w.
[76] Y. Chang, J. Liu, Y. Jiang, *et al.*, “Graph Fourier transform for spatial omics representation and analyses of complex organs,” *Res. Sq.*, p. rs.3.rs-3952048, Feb. 2024, doi: 10.21203/rs.3.rs-3952048/v1.
[77] C. E. Shannon, “A mathematical theory of communication,” *Bell Syst. Tech. J.*, vol. 27, no. 3, pp. 379–423, July 1948, doi: 10.1002/j.1538-7305.1948.tb01338.x.
[78] T. Gregor, D. W. Tank, E. F. Wieschaus, *et al.*, “Probing the Limits to Positional Information,” *Cell*, vol. 130, no. 1, pp. 153–164, July 2007, doi: 10.1016/j.cell.2007.05.025.
[79] F. Schaper, T. Jetka, and A. Dittrich, “Decoding cellular communication: An information theoretic perspective on cytokine and endocrine signaling,” *Curr. Opin. Endocr. Metab. Res.*, vol. 24, p. 100351, June 2022, doi: 10.1016/j.coemr.2022.100351.
[80] A. Karolak, S. Branciamore, J. S. McCune, *et al.*, “Concepts and Applications of Information Theory to Immuno-Oncology,” *Trends Cancer*, vol. 7, no. 4, pp. 335–346, Apr. 2021, doi: 10.1016/j.trecan.2020.12.013.
[81] A. C. Greenwald, N. G. Darnell, R. Hoefflin, *et al.*, “Integrative spatial analysis reveals a multi-layered organization of glioblastoma,” *Cell*, vol. 187, no. 10, pp. 2485-2501.e26, May 2024, doi: 10.1016/j.cell.2024.03.029.
[82] N. Tishby, F. C. Pereira, and W. Bialek, “The information bottleneck method,” Apr. 24, 2000, *arXiv*: arXiv:physics/0004057. doi: 10.48550/arXiv.physics/0004057.
[83] S. Dubnov, Z. Piran, H. Soreq, *et al.*, “Identifying maximally informative signal-aware representations of single-cell data using the Information Bottleneck,” July 17, 2024, *bioRxiv*. doi: 10.1101/2024.05.22.595292.
[84] N. Tishby and N. Zaslavsky, “Deep learning and the information bottleneck principle,” in *2015 IEEE Information Theory Workshop (ITW)*, Apr. 2015, pp. 1–5. doi: 10.1109/ITW.2015.7133169.

[85] X. Xu, S.-L. Huang, L. Zheng, *et al.*, "An Information Theoretic Interpretation to Deep Neural Networks," *Entropy*, vol. 24, no. 1, p. 135, Jan. 2022, doi: 10.3390/e24010135.
[86] J. Sohl-Dickstein, E. Weiss, N. Maheswaranathan, *et al.*, "Deep Unsupervised Learning using Nonequilibrium Thermodynamics," in *Proceedings of the 32nd International Conference on Machine Learning*, PMLR, June 2015, pp. 2256–2265. Available: https://proceedings.mlr.press/v37/sohl-dickstein15.html
[87] J. Ho, A. Jain, and P. Abbeel, "Denoising diffusion probabilistic models," in *Proceedings of the 34th International Conference on Neural Information Processing Systems*, in NIPS '20. Red Hook, NY, USA: Curran Associates Inc., Dec. 2020, pp. 6840–6851.
[88] "DALL·E 3." Accessed: Feb. 14, 2025. [Online]. Available: https://openai.com/index/dall-e-3/
[89] "Stability AI Image Models," Stability AI. Accessed: Feb. 14, 2025. [Online]. Available: https://stability.ai/stable-image
[90] Y. Song, J. Sohl-Dickstein, D. P. Kingma, *et al.*, "Score-Based Generative Modeling through Stochastic Differential Equations," Feb. 10, 2021, *arXiv*: arXiv:2011.13456. doi: 10.48550/arXiv.2011.13456.
[91] R. Rombach, A. Blattmann, D. Lorenz, *et al.*, "High-Resolution Image Synthesis with Latent Diffusion Models," in *2022 IEEE/CVF Conference on Computer Vision and Pattern Recognition (CVPR)*, June 2022, pp. 10674–10685. doi: 10.1109/CVPR52688.2022.01042.
[92] W. Peebles and S. Xie, "Scalable Diffusion Models with Transformers," in *2023 IEEE/CVF International Conference on Computer Vision (ICCV)*, Oct. 2023, pp. 4172–4182. doi: 10.1109/ICCV51070.2023.00387.
[93] K. Li, J. Li, Y. Tao, *et al.*, "stDiff: a diffusion model for imputing spatial transcriptomics through single-cell transcriptomics," *Brief. Bioinform.*, vol. 25, no. 3, p. bbae171, May 2024, doi: 10.1093/bib/bbae171.
[94] X. Li, F. Zhu, and W. Min, "SpaDiT: diffusion transformer for spatial gene expression prediction using scRNA-seq," *Brief. Bioinform.*, vol. 25, no. 6, p. bbae571, Nov. 2024, doi: 10.1093/bib/bbae571.
[95] S. Jiao, D. Lu, X. Zeng, *et al.*, "DiffuST: a latent diffusion model for spatial transcriptomics denoising," June 23, 2024, *bioRxiv*. doi: 10.1101/2024.06.19.599672.
[96] S. A. Khan, V. Lagani, R. Lehmann, *et al.*, "SpatialDiffusion: Predicting Spatial Transcriptomics with Denoising Diffusion Probabilistic Models," May 21, 2024, *bioRxiv*. doi: 10.1101/2024.05.21.595094.
[97] X. Li, W. Min, S. Wang, *et al.*, "stMCDI: Masked Conditional Diffusion Model with Graph Neural Network for Spatial Transcriptomics Data

Imputation," Mar. 16, 2024, *arXiv*: arXiv:2403.10863. doi: 10.48550/arXiv.2403.10863.
[98]L. Ambrogioni, "The Statistical Thermodynamics of Generative Diffusion Models: Phase Transitions, Symmetry Breaking, and Critical Instability," *Entropy*, vol. 27, no. 3, p. 291, Mar. 2025, doi: 10.3390/e27030291.
[99]G. Biroli and M. Mézard, "Generative diffusion in very large dimensions," *J. Stat. Mech. Theory Exp.*, vol. 2023, no. 9, p. 093402, Sept. 2023, doi: 10.1088/1742-5468/acf8ba.
[100]A. Sclocchi, A. Favero, and M. Wyart, "A phase transition in diffusion models reveals the hierarchical nature of data," *Proc. Natl. Acad. Sci.*, vol. 122, no. 1, p. e2408799121, Jan. 2025, doi: 10.1073/pnas.2408799121.
[101]U. Chitra, B. J. Arnold, H. Sarkar, *et al.*, "Mapping the topography of spatial gene expression with interpretable deep learning," *BioRxiv Prepr. Serv. Biol.*, p. 2023.10.10.561757, Oct. 2023, doi: 10.1101/2023.10.10.561757.
[102]R. A. Gatenby and E. T. Gawlinski, "A Reaction-Diffusion Model of Cancer Invasion," *Cancer Res.*, vol. 56, no. 24, pp. 5745–5753, Dec. 1996.
[103]H. J. M. Miniere, E. A. B. F. Lima, G. Lorenzo, *et al.*, "A mathematical model for predicting the spatiotemporal response of breast cancer cells treated with doxorubicin," *Cancer Biol. Ther.*, vol. 25, no. 1, p. 2321769, Dec. 2024, doi: 10.1080/15384047.2024.2321769.
[104]D. A. Hormuth, J. A. Weis, S. L. Barnes, *et al.*, "A mechanically coupled reaction–diffusion model that incorporates intra-tumoural heterogeneity to predict in vivo glioma growth," *J. R. Soc. Interface*, vol. 14, no. 128, p. 20161010, Mar. 2017, doi: 10.1098/rsif.2016.1010.
[105]M. Raissi, P. Perdikaris, and G. E. Karniadakis, "Physics-informed neural networks: A deep learning framework for solving forward and inverse problems involving nonlinear partial differential equations," *J. Comput. Phys.*, vol. 378, pp. 686–707, Feb. 2019, doi: 10.1016/j.jcp.2018.10.045.
[106]Q. Jiang and L. Wan, "A physics-informed neural SDE network for learning cellular dynamics from time-series scRNA-seq data," *Bioinformatics*, vol. 40, no. Supplement_2, pp. ii120–ii127, Sept. 2024, doi: 10.1093/bioinformatics/btae400.
[107]I. K. Boudjelthia, S. Milite, N. E. Kazwini, *et al.*, "NeuroVelo: interpretable learning of temporal cellular dynamics from single-cell data," June 10, 2024, *bioRxiv*. doi: 10.1101/2023.11.17.567500.

[108] H. Li, T. Ma, M. Hao, *et al.*, "Decoding functional cell–cell communication events by multi-view graph learning on spatial transcriptomics," *Brief. Bioinform.*, vol. 24, no. 6, p. bbad359, Nov. 2023, doi: 10.1093/bib/bbad359.
[109] B. Ji, X. Wang, D. Qiao, *et al.*, "SpaCCC: Large Language Model-Based Cell-Cell Communication Inference for Spatially Resolved Transcriptomic Data," *Big Data Min. Anal.*, vol. 7, no. 4, pp. 1129–1147, Dec. 2024, doi: 10.26599/BDMA.2024.9020056.
[110] Z. Liu, Y. Wang, S. Vaidya, *et al.*, "KAN: Kolmogorov–Arnold Networks," presented at the The Thirteenth International Conference on Learning Representations, Oct. 2024. Available: https://openreview.net/forum?id=Ozo7qJ5vZi
[111] A. Jamali, S. K. Roy, D. Hong, *et al.*, "How to Learn More? Exploring Kolmogorov–Arnold Networks for Hyperspectral Image Classification," *Remote Sens.*, vol. 16, no. 21, p. 4015, Jan. 2024, doi: 10.3390/rs16214015.
[112] A. Andersson and J. Lundeberg, "sepal: identifying transcript profiles with spatial patterns by diffusion-based modeling," *Bioinformatics*, vol. 37, no. 17, pp. 2644–2650, Sept. 2021, doi: 10.1093/bioinformatics/btab164.
[113] A. Massey, J. Stewart, C. Smith, *et al.*, "Mechanical properties of human tumour tissues and their implications for cancer development," *Nat. Rev. Phys.*, vol. 6, no. 4, pp. 269–282, Apr. 2024, doi: 10.1038/s42254-024-00707-2.
[114] Z. Mai, Y. Lin, P. Lin, *et al.*, "Modulating extracellular matrix stiffness: a strategic approach to boost cancer immunotherapy," *Cell Death Dis.*, vol. 15, no. 5, pp. 1–16, May 2024, doi: 10.1038/s41419-024-06697-4.
[115] M. Janiszewska, M. C. Primi, and T. Izard, "Cell adhesion in cancer: Beyond the migration of single cells," *J. Biol. Chem.*, vol. 295, no. 8, pp. 2495–2505, Feb. 2020, doi: 10.1074/jbc.REV119.007759.
[116] D. Wirtz, K. Konstantopoulos, and P. C. Searson, "The physics of cancer: the role of physical interactions and mechanical forces in metastasis," *Nat. Rev. Cancer*, vol. 11, no. 7, pp. 512–522, June 2011, doi: 10.1038/nrc3080.
[117] M. R. Zanotelli and C. A. Reinhart-King, "Mechanical Forces in Tumor Angiogenesis," *Adv. Exp. Med. Biol.*, vol. 1092, pp. 91–112, 2018, doi: 10.1007/978-3-319-95294-9_6.
[118] A. Hallou, R. He, B. D. Simons, *et al.*, "A computational pipeline for spatial mechano-transcriptomics," *Nat. Methods*, vol. 22, no. 4, pp. 737–750, Apr. 2025, doi: 10.1038/s41592-025-02618-1.

[119] A. J. Clevenger, M. K. McFarlin, J. P. M. Gorley, *et al.*, "Advances in cancer mechanobiology: Metastasis, mechanics, and materials," *APL Bioeng.*, vol. 8, no. 1, p. 011502, Mar. 2024, doi: 10.1063/5.0186042.
[120] B. Ladoux and R.-M. Mège, "Mechanobiology of collective cell behaviours," *Nat. Rev. Mol. Cell Biol.*, vol. 18, no. 12, pp. 743–757, Dec. 2017, doi: 10.1038/nrm.2017.98.
[121] F. Caforio, F. Regazzoni, S. Pagani, *et al.*, "Physics-informed neural network estimation of material properties in soft tissue nonlinear biomechanical models," *Comput. Mech.*, July 2024, doi: 10.1007/s00466-024-02516-x.
[122] T. Gao, R. Soldatov, H. Sarkar, *et al.*, "Haplotype-aware analysis of somatic copy number variations from single-cell transcriptomes," *Nat. Biotechnol.*, vol. 41, no. 3, pp. 417–426, Mar. 2023, doi: 10.1038/s41587-022-01468-y.
[123] C.-K. Mo, J. Liu, S. Chen, *et al.*, "Tumour evolution and microenvironment interactions in 2D and 3D space," *Nature*, vol. 634, no. 8036, pp. 1178–1186, Oct. 2024, doi: 10.1038/s41586-024-08087-4.
[124] A. P. Patel, I. Tirosh, J. J. Trombetta, *et al.*, "Single-cell RNA-seq highlights intratumoral heterogeneity in primary glioblastoma," *Science*, vol. 344, no. 6190, pp. 1396–1401, June 2014, doi: 10.1126/science.1254257.
[125] J. C. Rubinstein, S. Domanskyi, T. B. Sheridan, *et al.*, "Spatiotemporal Profiling Defines Persistence and Resistance Dynamics during Targeted Treatment of Melanoma," *Cancer Res.*, vol. 85, no. 5, pp. 987–1002, Mar. 2025, doi: 10.1158/0008-5472.CAN-24-0690.
[126] F. Liu, F. Shi, F. Du, *et al.*, "CoT: a transformer-based method for inferring tumor clonal copy number substructure from scDNA-seq data," *Brief. Bioinform.*, vol. 25, no. 3, p. bbae187, May 2024, doi: 10.1093/bib/bbae187.
[127] C. Ma, M. Balaban, J. Liu, *et al.*, "Inferring allele-specific copy number aberrations and tumor phylogeography from spatially resolved transcriptomics," *Nat. Methods*, pp. 1–9, Oct. 2024, doi: 10.1038/s41592-024-02438-9.
[128] J. A. Rodrigues, "Using Physics-Informed Neural Networks (PINNs) for Tumor Cell Growth Modeling," *Mathematics*, vol. 12, no. 8, Art. no. 8, Jan. 2024, doi: 10.3390/math12081195.
[129] Y. Fu, A. W. Jung, R. V. Torne, *et al.*, "Pan-cancer computational histopathology reveals mutations, tumor composition and prognosis," *Nat. Cancer*, vol. 1, no. 8, pp. 800–810, Aug. 2020, doi: 10.1038/s43018-020-0085-8.

[130] S. Farahmand, A. I. Fernandez, F. S. Ahmed, *et al.*, “Deep learning trained on hematoxylin and eosin tumor region of Interest predicts HER2 status and trastuzumab treatment response in HER2+ breast cancer,” *Mod. Pathol.*, vol. 35, no. 1, pp. 44–51, Jan. 2022, doi: 10.1038/s41379-021-00911-w.
[131] K. Clifton, M. Anant, G. Aihara, *et al.*, “STalign: Alignment of spatial transcriptomics data using diffeomorphic metric mapping,” *Nat. Commun.*, vol. 14, no. 1, p. 8123, Dec. 2023, doi: 10.1038/s41467-023-43915-7.
[132] C. R. Harris, E. T. McKinley, J. T. Roland, *et al.*, “Quantifying and correcting slide-to-slide variation in multiplexed immunofluorescence images,” *Bioinformatics*, vol. 38, no. 6, pp. 1700–1707, Mar. 2022, doi: 10.1093/bioinformatics/btab877.
[133] P. V. Kharchenko, L. Silberstein, and D. T. Scadden, “Bayesian approach to single-cell differential expression analysis,” *Nat. Methods*, vol. 11, no. 7, pp. 740–742, July 2014, doi: 10.1038/nmeth.2967.
[134] Y. Shan, Q. Zhang, W. Guo, *et al.*, “TIST: Transcriptome and Histopathological Image Integrative Analysis for Spatial Transcriptomics,” *Genomics Proteomics Bioinformatics*, vol. 20, no. 5, pp. 974–988, Oct. 2022, doi: 10.1016/j.gpb.2022.11.012.
[135] S. Domanskyi, A. Srivastava, J. Kaster, *et al.*, “Nextflow pipeline for Visium and H&E data from patient-derived xenograft samples,” *Cell Rep. Methods*, vol. 4, no. 5, p. 100759, May 2024, doi: 10.1016/j.crmeth.2024.100759.
[136] Y. Long, K. S. Ang, R. Sethi, *et al.*, “Deciphering spatial domains from spatial multi-omics with SpatialGlue,” *Nat. Methods*, vol. 21, no. 9, pp. 1658–1667, Sept. 2024, doi: 10.1038/s41592-024-02316-4.
[137] W. Tang, H. Wen, R. Liu, *et al.*, “Single-Cell Multimodal Prediction via Transformers,” in *Proceedings of the 32nd ACM International Conference on Information and Knowledge Management*, Oct. 2023, pp. 2422–2431. doi: 10.1145/3583780.3615061.
[138] D. Haviv, J. Remšík, M. Gatie, *et al.*, “The covariance environment defines cellular niches for spatial inference,” *Nat. Biotechnol.*, vol. 43, no. 2, pp. 269–280, Feb. 2025, doi: 10.1038/s41587-024-02193-4.
[139] A. Vaidya, A. Zhang, G. Jaume, *et al.*, “Molecular-driven Foundation Model for Oncologic Pathology,” Jan. 28, 2025, *arXiv*: arXiv:2501.16652. doi: 10.48550/arXiv.2501.16652.
[140] G. Wang, W. Chen, P. Zhang, *et al.*, “A visual–omics foundation model to bridge histopathology image with transcriptomics,” Apr. 16, 2025, *Research Square*. doi: 10.21203/rs.3.rs-5183775/v1.
[141] C. Zhang, D. Song, C. Huang, *et al.*, “Heterogeneous Graph Neural Network,” in *Proceedings of the 25th ACM SIGKDD International*

Conference on Knowledge Discovery & Data Mining, in KDD ’19. New York, NY, USA: Association for Computing Machinery, July 2019, pp. 793–803. doi: 10.1145/3292500.3330961.
[142] C. Zuo, J. Xia, and L. Chen, “Dissecting tumor microenvironment from spatially resolved transcriptomics data by heterogeneous graph learning,” *Nat. Commun.*, vol. 15, no. 1, p. 5057, June 2024, doi: 10.1038/s41467-024-49171-7.
[143] R. J. Chen, C. Chen, Y. Li, *et al.*, “Scaling Vision Transformers to Gigapixel Images via Hierarchical Self-Supervised Learning,” in *2022 IEEE/CVF Conference on Computer Vision and Pattern Recognition (CVPR)*, June 2022, pp. 16123–16134. doi: 10.1109/CVPR52688.2022.01567.
[144] E. Haber, A. Deshpande, J. Ma, *et al.*, “Unified integration of spatial transcriptomics across platforms,” Apr. 21, 2025, *bioRxiv*. doi: 10.1101/2025.03.31.646238.
[145] H. Xu, N. Usuyama, J. Bagga, *et al.*, “A whole-slide foundation model for digital pathology from real-world data,” *Nature*, vol. 630, no. 8015, pp. 181–188, June 2024, doi: 10.1038/s41586-024-07441-w.
[146] F. Mahmood, “A benchmarking crisis in biomedical machine learning,” *Nat. Med.*, vol. 31, no. 4, pp. 1060–1060, Apr. 2025, doi: 10.1038/s41591-025-03637-3.
[147] G. Campanella, S. Chen, M. Singh, *et al.*, “A clinical benchmark of public self-supervised pathology foundation models,” *Nat. Commun.*, vol. 16, no. 1, p. 3640, Apr. 2025, doi: 10.1038/s41467-025-58796-1.
[148] A. Zhang, G. Jaume, A. Vaidya, *et al.*, “Accelerating Data Processing and Benchmarking of AI Models for Pathology,” Feb. 10, 2025, *arXiv*: arXiv:2502.06750. doi: 10.48550/arXiv.2502.06750.
[149] K. Meng-Lin, C.-Y. Ung, C. Zhang, *et al.*, “SPIN-AI: A Deep Learning Model That Identifies Spatially Predictive Genes,” *Biomolecules*, vol. 13, no. 6, p. 895, May 2023, doi: 10.3390/biom13060895.
[150] “MOSAIC is the world’s largest spatial multiomics dataset in oncology.” Accessed: Feb. 14, 2025. [Online]. Available: https://www.mosaic-research.com/
[151] B. L. Angarola, S. Sharma, N. Katiyar, *et al.*, “Comprehensive single-cell aging atlas of healthy mammary tissues reveals shared epigenomic and transcriptomic signatures of aging and cancer,” *Nat. Aging*, vol. 5, no. 1, pp. 122–143, Jan. 2025, doi: 10.1038/s43587-024-00751-8.
[152] M. A. Bogue, G. A. Churchill, and E. J. Chesler, “Collaborative Cross and Diversity Outbred data resources in the Mouse Phenome Database,” *Mamm. Genome*, vol. 26, no. 9, pp. 511–520, Oct. 2015, doi: 10.1007/s00335-015-9595-6.

[153]D. A. Skelly, J. P. Graham, M. Cheng, *et al.*, "Mapping the genetic landscape establishing a tumor immune microenvironment favorable for anti-PD-1 response," *Cell Rep.*, vol. 44, no. 5, May 2025, doi: 10.1016/j.celrep.2025.115698.
[154]C. O'Connor, G. R. Keele, W. Martin, *et al.*, "Unraveling the genetics of arsenic toxicity with cellular morphology QTL," *PLOS Genet.*, vol. 20, no. 4, p. e1011248, Apr. 2024, doi: 10.1371/journal.pgen.1011248.
[155]X. Liu, Q. Shen, and S. Zhang, "Cross-species cell-type assignment from single-cell RNA-seq data by a heterogeneous graph neural network," *Genome Res.*, vol. 33, no. 1, pp. 96–111, Jan. 2023, doi: 10.1101/gr.276868.122.
[156]B. Zhang, S. Zhang, and S. Zhang, "Whole brain alignment of spatial transcriptomics between humans and mice with BrainAlign," *Nat. Commun.*, vol. 15, no. 1, p. 6302, July 2024, doi: 10.1038/s41467-024-50608-2.
[157]T. Shay, V. Jojic, O. Zuk, *et al.*, "Conservation and divergence in the transcriptional programs of the human and mouse immune systems," *Proc. Natl. Acad. Sci.*, vol. 110, no. 8, pp. 2946–2951, Feb. 2013, doi: 10.1073/pnas.1222738110.
[158]P. Mukashyaka, T. B. Sheridan, A. Foroughi pour, *et al.*, "SAMPLER: unsupervised representations for rapid analysis of whole slide tissue images," *eBioMedicine*, vol. 99, p. 104908, Jan. 2024, doi: 10.1016/j.ebiom.2023.104908.
[159]J. Kim, S. Rustam, J. M. Mosquera, *et al.*, "Unsupervised discovery of tissue architecture in multiplexed imaging," *Nat. Methods*, vol. 19, no. 12, pp. 1653–1661, Dec. 2022, doi: 10.1038/s41592-022-01657-2.
[160]Y. Lin, Y. Wang, Y. Liang, *et al.*, "Sampling and ranking spatial transcriptomics data embeddings to identify tissue architecture," *Front. Genet.*, vol. 13, Aug. 2022, doi: 10.3389/fgene.2022.912813.
[161]Z. Wu, A. E. Trevino, E. Wu, *et al.*, "Graph deep learning for the characterization of tumour microenvironments from spatial protein profiles in tissue specimens," *Nat. Biomed. Eng.*, vol. 6, no. 12, pp. 1435–1448, Dec. 2022, doi: 10.1038/s41551-022-00951-w.
[162]W. Yang, P. Wang, S. Xu, *et al.*, "Deciphering cell–cell communication at single-cell resolution for spatial transcriptomics with subgraph-based graph attention network," *Nat. Commun.*, vol. 15, no. 1, p. 7101, Aug. 2024, doi: 10.1038/s41467-024-51329-2.

Figure

Click here to access/download;Figure;2025_Research_Chuang_AI_1.png

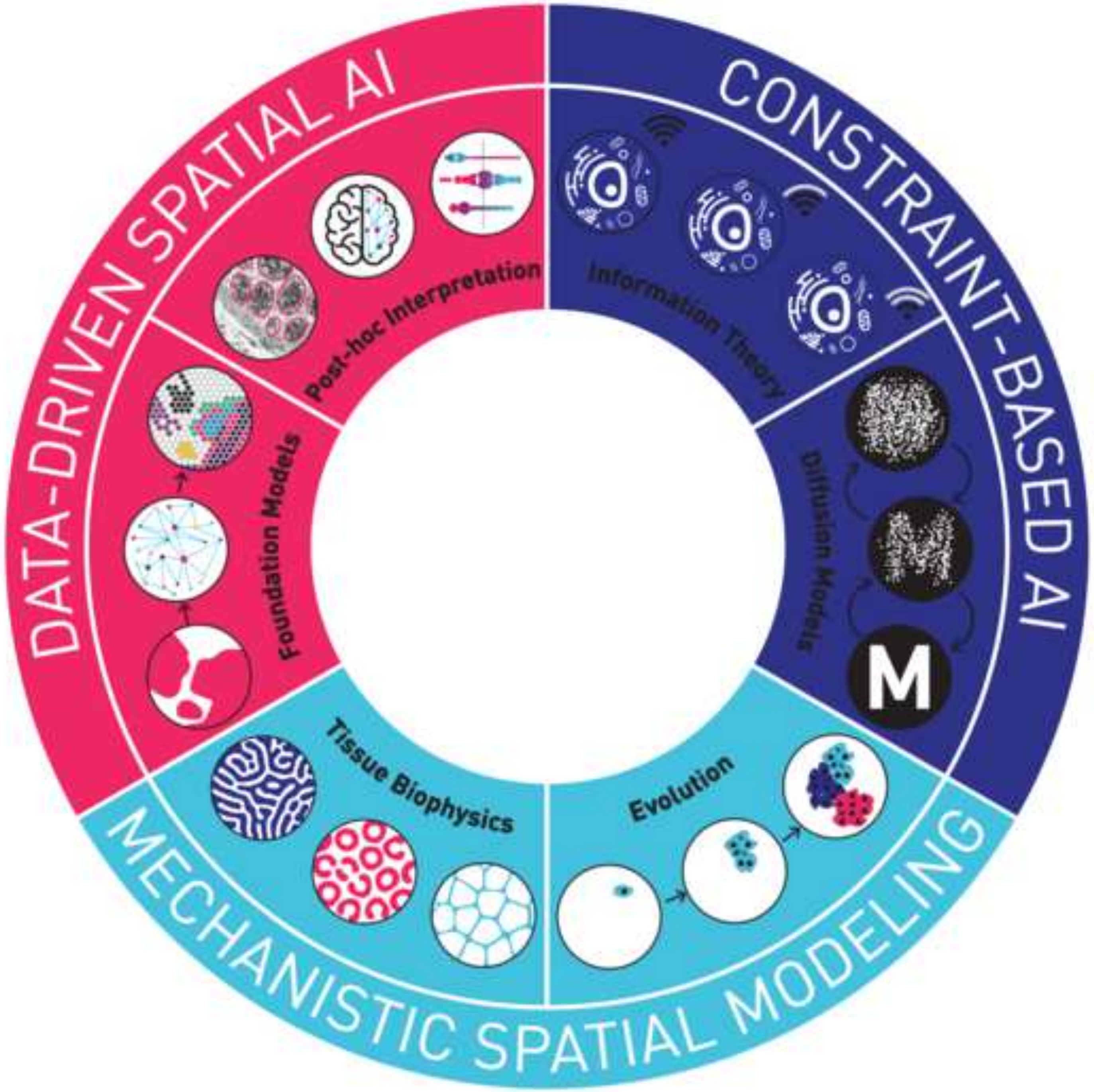

Response to reviewers Click here to access/download;Personal Cover;response-to-reviewers.docx

Dear GigaScience,

We would like to thank the reviewers and editor for generously taking the time to consider this manuscript. The constructive feedback has helped us improve the work. Point-by-point responses to each of the reviewer comments are provided below.

Best regards,

Jeff Chuang

Reviewer reports:
Reviewer #1: This is a very nice review providing a new and original angle to the present stat e of combining AI-based analysis approaches with spatially resolved omics methods (brrimarily proteomics and transcriptomics). The review also points to new directions of development, and is truly inspirational in its outlooks and future perspectives, which is appreciated. The text is well-written and easy to follow, though at points a bit brief in methods descriptions, as can be expected from this type of broad review paper. I was missing references in the introduction, feeling that many of the statements would have benefitted from pointing to e.g. other review papers in the field. Adding more references to the introduction would improve the text and also make the review more useful for an audience without expert knowledge in spatially resolved omics.

We thank the reviewer for their comment and agree that sufficient references were lacking from our introduction. We added several references to the introduction of the paper most of which are review papers. We also noticed that in other chapters, concepts such as diffusion models and information theory lacked references. We have added references for those as well.

Abbreviations are thoroughly described apart from 'patient IRB', which should be defined, or perhaps re-phrased to not use an abbreviation.

We added the IRB abbreviation to the end of the manuscript.

Reviewer #2: 1. The manuscript outlines three emerging paradigms (data-driven, constraint-based, and mechanistic spatial AI) but lacks a rigorous critical analysis of their practical limitations in cancer research. For data-driven foundation models, while post-hoc interpretation methods (e.g., SHAP, LIME) are mentioned, the review does not sufficiently address their inherent weaknesses—such as the inability to establish causal relationships or the risk of overfitting to batch effects in heterogeneous spatial omics datasets.

We thank the reviewer for their comment. We agree that the way we wrote the text could confuse the reader about the link between the post-hoc interpretation and causal analysis. We have added the following paragraph to clarify this point:

“A limitation of post-hoc interpretation approaches is that they reveal associations rather than causal relationships, making them more suited for hypothesis generation. These approaches can inadvertently attribute importance to spurious confounders and batch effects, so their use should be accompanied by batch correction, and ideally, validation on external datasets.”

Similarly, constraint-based models (e.g., diffusion models) are noted for integrating spatial hierarchies, but there is little discussion of how their performance scales with high-dimensional spatial proteomics data (e.g., >1000 markers) or their susceptibility to artifacts from variable antibody panels.

Current antibody-based proteomics data tends to be low-dimensional (on the order of 10-100 protein). High-dimensional protein data (>1000 markers) of the sort the reviewer mentions tend to be masspec-based (e.g. MALDI), and such data have challenges similar to spatial transcriptomics. We have now mentioned these challenges for high dimensional spatial omics data in the text and proposed latent diffusion models as a solution (e.g. DiTs). Some methods using this approach are already mentioned in the text (e.g. SpaDiT, stDiff, DiffuST). However, we recognize that the text may not have been clear enough in linking these ideas. We added the following to the text:

"The large number of markers in spatial omics poses a challenge for diffusion models. Embedding data into a lower dimensional space reduces noise, leverages marker correlations, and makes high-dimensional data more manageable. This latent diffusion model approach has been successfully applied to spatial transcriptomics data in methods such as stDiff [1] and SpaDiT [2] which utilize DiTs to impute missing genes, and may be useful for other high dimensional omics types such as mass spectrometry as well. "

Mechanistic models like PINNs are highlighted, yet the manuscript overlooks critical barriers such as the scarcity of longitudinal spatial data in clinical cancer samples, which limits their training and validation. A more balanced assessment of these limitations is essential for readers to gauge the current translational readiness of each paradigm.

We thank the reviewer for this comment. We note that some of the limitations of PINNs are mentioned in the text already:

"These methods require dynamical data for training, and they have been applied to scRNA data to predict cell state dynamics [3] and for RNA velocity inference [4]. So far, application of PINNs to tissues has been limited due to scarcity of time-course data. However, as longitudinal SRO data improve, PINNs can provide a framework to infer interpretable physical processes and parameters from them."

However, even without time-series data these frameworks are valuable, a point which may not have been sufficiently clear. We therefore added the following sentences to the text:

"Nevertheless, PINNs can still be valuably applied to static data for diffusive systems at steady-state or quasi-steady state, where timescales are sufficiently separated. Methods developed with this philosophy already exist and may be adaptable to PINNs."

This provides more context for the prior text, which follows immediately afterward:
"For example, HoloNet [5] is a graph neural network that uses ligand diffusion equations to infer ligand-receptor interactions from spatial transcriptomics data, and SpaCCC [6] integrates this into a transformer framework. These two methods overcome the limitations of time-course scarcity by stripping chemical diffusion and reaction equations from their dynamics and treating them as steady-state functional forms."

2. The review emphasizes the importance of multi-modal data integration (e.g., H&E, spatial transcriptomics, proteomics) but fails to address key technical bottlenecks in achieving robust integration. For instance, while methods like SpatialGlue and OmiCLIP are cited, there is little discussion of how to resolve fundamental inconsistencies: varying spatial resolutions (0.25-100 microns across technologies),

We added the following paragraph to cover the issue of ‘varying spatial resolutions’ and proposed some approaches to address it:

“Different spatial omics modalities from the same tissue often have mismatched resolution, complicating integration. Heterogenous GNNs [7], which assign modality-specific attributes to nodes, have been used to study the tumor microenvironment in spatial transcriptomics data [8] and could potentially support multi-resolution integration by linking nodes (e.g. cells or spots) by spatial proximity. Similarly, multi-resolution transformers have shown success in histopathology [9] by jointly processing images at multiple zoom scales. A similar strategy could be adapted for spatial omics integration by treating each resolution as a distinct modality.”

platform-specific noise (e.g., dropout in spatial RNA), and divergent marker panels in proteomics. This gap hinders understanding of the current feasibility of holistic spatial profiling.

We agree this is an important topic. We refer the reviewer to the ‘Spatial omics foundation models’ section which we believe addresses their concern about divergent marker panels:

“However, integrating protein data across experiments remains challenging due to variations in protein panels and measured antibody intensity distributions. These issues may be alleviated by encoding protein data into more robust representations, e.g. by non-negative matrix factorization [10] or generative neural networks, which have also enabled powerful capabilities such as combinatorial protein signal decomposition [11] [12]. A promising recent method named KRONOS [13] addresses the issue of protein marker heterogeneity by an innovative tokenization approach.”

Still, as the reviewer correctly points out, this topic could also be perceived as a data integration challenge. We added the following to the ‘data integration’ section to directly address this issue:

“Platform-specific noise (e.g. spatial RNA dropout) further complicates integration. Methods adapted from single-cell analysis can help mitigate this issue. For example, LLOKI [14] combines neighborhood and distributional similarity and pretrained single-cell foundation models to reduce expression sparsity and correct batch effects across datasets. Divergent marker panels, particularly in spatial proteomics, also impede integration. This can be mitigated by recognizing that phenotypes often recur even when exact markers differ. Integration with other data types (e.g. H&E) and marker-agnostic encoding methods [13] can be effective as well. “

Additionally, despite noting mouse models as critical for mechanistic validation, the manuscript underdeveloped the challenge of cross-species translation. While BrainAlign and Nicheformer are mentioned, there is insufficient analysis of how species-specific differences in tissue architecture (e.g., immune microenvironment organization) or gene expression dynamics might invalidate model

transferability. Without addressing these, the review risks overstating the practical utility of current integration frameworks.

We thank the reviewer for their comment and added the following caveat to the text:

"Despite evolutionary similarities, human and mouse may diverge in many aspects of tissue architecture including fine immune microenvironmental organization and gene expression dynamics [15]. Computational mapping of tissue architecture across the two species is a broad challenge whose solution would improve the translational use of mouse models for human cancer research."

3. The manuscript acknowledges that spatial omics foundation models lack standardized benchmarking but does not sufficiently elaborate on this critical gap. For histopathology foundation models, tumor-type classification is noted as a common benchmark, but the review does not propose how to evaluate more complex tasks essential for cancer research—such as distinguishing fine-grained tumor microenvironments (e.g., immune-hot vs. immune-excluded regions) or predicting treatment response from spatial patterns. Similarly, for spatial transcriptomics/proteomics models, there is no discussion of reference datasets, gold-standard metrics (beyond clustering or imputation accuracy), or inter-lab reproducibility challenges. This omission is problematic because without agreed-upon benchmarks, progress in the field risks being fragmented and difficult to compare. The review would benefit from proposing a roadmap for community-driven standardization, including multi-scale evaluation tasks and validation against clinical endpoints (e.g., patient survival).

We added a new subsection titled 'Model benchmarking' to the 'Data consideration' section to address this issue:

"Rapid progress in digital pathology foundation models make rigorous benchmarking essential. Current *de facto* H&E benchmarks, such as TCGA subtype classification and molecular prediction [16], [17], largely reflect historical data availability rather than a coordinated community-driven effort aimed at biological discovery and translational impact [18]. To close this gap, the field needs community initiatives to curate agreed-upon public datasets and standardize metrics. While clinically grounded datasets and benchmarks are emerging [19], [20], broad institutional momentum remains limited; nonetheless, we anticipate increasing standardization in the coming years.

In the SRO domain the need is even more acute, as limited large-scale datasets and rapidly evolving assays make benchmarking ambiguous. Existing benchmarks mostly target unsupervised tasks (e.g. spatial domain identification) or self-supervised objectives (e.g. imputing missing genes), or rely on expert-annotated datasets that are small and domain-specific [21]. Where labels exist, they are typically intra-tissue (e.g. cell type annotations) rather than cross-sample, and patient-level annotation is scarce. These benchmarks are valuable for research, but to build parallels to the H&E AI models would require clinically grounded benchmarks built on large-scale patient-level annotations. Organizing consortia to generate clinically annotated, patient-derived spatial omics datasets is essential to establish relevant ground truth and enable clinically meaningful benchmarking."

References

[1] "stDiff: a diffusion model for imputing spatial transcriptomics through single-cell transcriptomics | Briefings in Bioinformatics | Oxford Academic." Accessed: Oct. 02, 2024. [Online]. Available: https://academic.oup.com/bib/article/25/3/bbae171/7646375
[2] X. Li, F. Zhu, and W. Min, "SpaDiT: Diffusion Transformer for Spatial Gene Expression Prediction using scRNA-seq," July 18, 2024, *arXiv*: arXiv:2407.13182. doi: 10.48550/arXiv.2407.13182.
[3] Q. Jiang and L. Wan, "A physics-informed neural SDE network for learning cellular dynamics from time-series scRNA-seq data," *Bioinformatics*, vol. 40, no. Supplement_2, pp. ii120–ii127, Sept. 2024, doi: 10.1093/bioinformatics/btae400.
[4] I. K. Boudjelthia, S. Milite, N. E. Kazwini, Y. Huang, A. Sottoriva, and G. Sanguinetti, "NeuroVelo: interpretable learning of temporal cellular dynamics from single-cell data," June 10, 2024, *bioRxiv*. doi: 10.1101/2023.11.17.567500.
[5] H. Li *et al.*, "Decoding functional cell–cell communication events by multi-view graph learning on spatial transcriptomics," *Brief. Bioinform.*, vol. 24, no. 6, p. bbad359, Nov. 2023, doi: 10.1093/bib/bbad359.
[6] B. Ji, L. Xu, and S. Peng, "SpaCCC: Large language model-based cell-cell communication inference for spatially resolved transcriptomic data," Feb. 23, 2024, *bioRxiv*. doi: 10.1101/2024.02.21.581369.
[7] C. Zhang, D. Song, C. Huang, A. Swami, and N. V. Chawla, "Heterogeneous Graph Neural Network," in *Proceedings of the 25th ACM SIGKDD International Conference on Knowledge Discovery & Data Mining*, in KDD '19. New York, NY, USA: Association for Computing Machinery, July 2019, pp. 793–803. doi: 10.1145/3292500.3330961.
[8] C. Zuo, J. Xia, and L. Chen, "Dissecting tumor microenvironment from spatially resolved transcriptomics data by heterogeneous graph learning," *Nat. Commun.*, vol. 15, no. 1, p. 5057, June 2024, doi: 10.1038/s41467-024-49171-7.
[9] "[2206.02647] Scaling Vision Transformers to Gigapixel Images via Hierarchical Self-Supervised Learning." Accessed: Aug. 22, 2025. [Online]. Available: https://arxiv.org/abs/2206.02647
[10] A. F. Pour *et al.*, "Prediction of Outcome from Spatial Protein Profiling of Triple-Negative Breast Cancers," Apr. 18, 2025, *bioRxiv*. doi: 10.1101/2025.04.18.649541.
[11] R. Ben-Uri *et al.*, "High-dimensional imaging using combinatorial channel multiplexing and deep learning," *Nat. Biotechnol.*, pp. 1–14, Mar. 2025, doi: 10.1038/s41587-025-02585-0.
[12] S. Ayub, H. W. Jackson, A. Selega, and K. R. Campbell, "Multi-view deep learning of highly multiplexed imaging data improves association of cell states with clinical outcomes," Mar. 17, 2025, *bioRxiv*. doi: 10.1101/2025.03.14.643377.
[13] M. Shaban *et al.*, "A Foundation Model for Spatial Proteomics," June 05, 2025, *arXiv*: arXiv:2506.03373. doi: 10.48550/arXiv.2506.03373.
[14] E. Haber, A. Deshpande, J. Ma, and S. Krieger, "Unified integration of spatial transcriptomics across platforms," Apr. 21, 2025, *bioRxiv*. doi: 10.1101/2025.03.31.646238.
[15] T. Shay *et al.*, "Conservation and divergence in the transcriptional programs of the human and mouse immune systems," *Proc. Natl. Acad. Sci.*, vol. 110, no. 8, pp. 2946–2951, Feb. 2013, doi: 10.1073/pnas.1222738110.
[16] R. J. Chen *et al.*, "Towards a general-purpose foundation model for computational pathology," *Nat. Med.*, vol. 30, no. 3, pp. 850–862, Mar. 2024, doi: 10.1038/s41591-024-02857-3.
[17] H. Xu *et al.*, "A whole-slide foundation model for digital pathology from real-world data," *Nature*, vol. 630, no. 8015, pp. 181–188, June 2024, doi: 10.1038/s41586-024-07441-w.
[18] F. Mahmood, "A benchmarking crisis in biomedical machine learning," *Nat. Med.*, vol. 31, no. 4, pp. 1060–1060, Apr. 2025, doi: 10.1038/s41591-025-03637-3.
[19] G. Campanella *et al.*, "A clinical benchmark of public self-supervised pathology foundation models," *Nat. Commun.*, vol. 16, no. 1, p. 3640, Apr. 2025, doi: 10.1038/s41467-025-58796-1.

[20] A. Zhang, G. Jaume, A. Vaidya, T. Ding, and F. Mahmood, “Accelerating Data Processing and Benchmarking of AI Models for Pathology,” Feb. 10, 2025, *arXiv*: arXiv:2502.06750. doi: 10.48550/arXiv.2502.06750.

[21] R. Zahedi *et al.*, “Deep learning in spatially resolved transcriptomics: a comprehensive technical view,” *Brief. Bioinform.*, vol. 25, no. 2, p. bbae082, Mar. 2024, doi: 10.1093/bib/bbae082.
