# Supplementary material for: Emerging AI approaches for cancer spatial omics
Source: Gigascience. 2025 Oct 16;14:giaf128. doi: 10.1093/gigascience/giaf128 (PMC12612624; doi:10.1093/gigascience/giaf128)
Supplement: giaf128_Authors_Response_To_Reviewer_Comments_Original_Submission [file giaf128_authors_response_to_reviewer_comments_original_submission.pdf]

Author's Response To Reviewer Comments

Note: We have provided a file describing all responses, described as "Personal Cover." The content is identical to the material below, but the responses are marked in red to more clearly separate the reviewer comments and responses.

Dear GigaScience,
We would like to thank the reviewers and editor for generously taking the time to consider this manuscript. The constructive feedback has helped us improve the work. Point-by-point responses to each of the reviewer comments are provided below.

Best regards,
Jeff Chuang

Reviewer reports:
Reviewer #1.1: This is a very nice review providing a new and original angle to the present stat e of combining AI-based analysis approaches with spatially resolved omics methods (brrimarily proteomics and transcriptomics). The review also points to new directions of development, and is truly inspirational in its outlooks and future perspectives, which is appreciated. The text is well-written and easy to follow, though at points a bit brief in methods descriptions, as can be expected from this type of broad review paper. I was missing references in the introduction, feeling that many of the statements would have benefitted from pointing to e.g. other review papers in the field. Adding more references to the introduction would improve the text and also make the review more useful for an audience without expert knowledge in spatially resolved omics.

Response: We thank the reviewer for their comment and agree that sufficient references were lacking from our introduction. We added several references to the introduction of the paper most of which are review papers. We also noticed that in other chapters, concepts such as diffusion models and information theory lacked references. We have added references for those as well.

2. Abbreviations are thoroughly described apart from 'patient IRB', which should be defined, or perhaps re-phrased to not use an abbreviation.

Response: We added the IRB abbreviation to the end of the manuscript.

Reviewer #2.1a: The manuscript outlines three emerging paradigms (data-driven, constraint-based, and mechanistic spatial AI) but lacks a rigorous critical analysis of their practical limitations in cancer research. For data-driven foundation models, while post-hoc interpretation methods (e.g., SHAP, LIME) are mentioned, the review does not sufficiently address their inherent weaknesses—such as the inability to establish causal relationships or the risk of overfitting to batch effects in heterogeneous spatial omics datasets.

Response: We thank the reviewer for their comment. We agree that the way we wrote the text could confuse the reader about the link between the post-hoc interpretation and causal analysis. We have added the following paragraph to clarify this point:

“A limitation of post-hoc interpretation approaches is that they reveal associations rather than causal relationships, making them more suited for hypothesis generation. These approaches can inadvertently attribute importance to spurious confounders and batch effects, so their use should be accompanied by batch correction, and ideally, validation on external datasets.”

1b: Similarly, constraint-based models (e.g., diffusion models) are noted for integrating spatial hierarchies, but there is little discussion of how their performance scales with high-dimensional spatial proteomics data (e.g., >1000 markers) or their susceptibility to artifacts from variable antibody panels.

Response: Current antibody-based proteomics data tends to be low-dimensional (on the order of 10-100 protein). High-dimensional protein data (>1000 markers) of the sort the reviewer mentions tend to be masspec-based (e.g. MALDI), and such data have challenges similar to spatial transcriptomics. We have now mentioned these challenges for high dimensional spatial omics data in the text and proposed latent diffusion models as a solution (e.g. DiTs). Some methods using this approach are already mentioned in the text (e.g. SpaDiT, stDiff, DiffuST). However, we recognize that the text may not have been clear enough in linking these ideas. We added the following to the text:
“The large number of markers in spatial omics poses a challenge for diffusion models. Embedding data into a lower dimensional space reduces noise, leverages marker correlations, and makes high-dimensional data more manageable. This latent diffusion model approach has been successfully applied to spatial transcriptomics data in methods such as stDiff [1] and SpaDiT [2] which utilize DiTs to impute missing genes, and may be useful for other high dimensional omics types such as mass spectrometry as well. “

1c: Mechanistic models like PINNs are highlighted, yet the manuscript overlooks critical barriers such as the scarcity of longitudinal spatial data in clinical cancer samples, which limits their training and validation. A more balanced assessment of these limitations is essential for readers to gauge the current translational readiness of each paradigm.

Response: We thank the reviewer for this comment. We note that some of the limitations of PINNs are mentioned in the text already:

“These methods require dynamical data for training, and they have been applied to scRNA data to predict cell state dynamics [3] and for RNA velocity inference [4]. So far, application of PINNs to tissues has been limited due to scarcity of time-course data. However, as longitudinal SRO data improve, PINNs can provide a framework to infer interpretable physical processes and parameters from them.”

However, even without time-series data these frameworks are valuable, a point which may not have been sufficiently clear. We therefore added the following sentences to the text:
“Nevertheless, PINNs can still be valuably applied to static data for diffusive systems at steady-state or quasi-steady state, where timescales are sufficiently separated. Methods developed with this philosophy already exist and may be adaptable to PINNs.”
This provides more context for the prior text, which follows immediately afterward:
“For example, HoloNet [5] is a graph neural network that uses ligand diffusion equations to infer ligand-receptor interactions from spatial transcriptomics data, and SpaCCC [6] integrates this into a transformer framework. These two methods overcome the limitations of time-course scarcity by stripping chemical diffusion and reaction equations from their dynamics and treating them as steady-state functional forms.”

2a: The review emphasizes the importance of multi-modal data integration (e.g., H&E, spatial transcriptomics, proteomics) but fails to address key technical bottlenecks in achieving robust integration. For instance, while methods like SpatialGlue and OmiCLIP are cited, there is little discussion of how to resolve fundamental inconsistencies: varying spatial resolutions (0.25-100 microns across technologies)...

Response: We added the following paragraph to cover the issue of ‘varying spatial resolutions’ and proposed some approaches to address it:
“Different spatial omics modalities from the same tissue often have mismatched resolution, complicating integration. Heterogenous GNNs [7], which assign modality-specific attributes to nodes, have been used to study the tumor microenvironment in spatial transcriptomics data [8] and could potentially support multi-resolution integration by linking nodes (e.g. cells or spots) by spatial proximity. Similarly, multi-resolution transformers have shown success in histopathology [9] by jointly processing images at multiple zoom scales. A similar strategy could be adapted for spatial omics integration by treating each resolution as a distinct modality.”

2b: ...platform-specific noise (e.g., dropout in spatial RNA), and divergent marker panels in proteomics. This gap hinders understanding of the current feasibility of holistic spatial profiling.

Response: We agree this is an important topic. We refer the reviewer to the ‘Spatial omics foundation models’ section which we believe addresses their concern about divergent marker panels:

“However, integrating protein data across experiments remains challenging due to variations in protein panels and measured antibody intensity distributions. These issues may be alleviated by encoding protein data into more robust representations, e.g. by non-negative matrix factorization [10] or generative neural networks, which have also enabled powerful capabilities such as combinatorial protein signal decomposition [11] [12]. A promising recent method named KRONOS [13] addresses the issue of protein marker heterogeneity by an innovative tokenization approach.”

Still, as the reviewer correctly points out, this topic could also be perceived as a data integration challenge. We added the following to the ‘data integration’ section to directly address this issue:
“Platform-specific noise (e.g. spatial RNA dropout) further complicates integration. Methods adapted from single-cell analysis can help mitigate this issue. For example, LLOKI [14] combines neighborhood and distributional similarity and pretrained single-cell foundation models to reduce expression sparsity and correct batch effects across datasets. Divergent marker panels, particularly in spatial proteomics, also impede integration. This can be mitigated by recognizing that phenotypes often recur even when exact markers differ. Integration with other data types (e.g. H&E) and marker-agnostic encoding methods [13] can be effective as well. “

2c: Additionally, despite noting mouse models as critical for mechanistic validation, the manuscript underdeveloped the challenge of cross-species translation. While BrainAlign and Nicheformer are mentioned, there is insufficient analysis of how species-specific differences in tissue architecture (e.g., immune microenvironment organization) or gene expression dynamics might invalidate model transferability. Without addressing these, the review risks overstating the practical utility of current integration frameworks.

Response: We thank the reviewer for their comment and added the following caveat to the text:
“Despite evolutionary similarities, human and mouse may diverge in many aspects of tissue architecture including fine immune microenvironmental organization and gene expression dynamics [15]. Computational mapping of tissue architecture across the two species is a broad challenge whose solution would improve the translational use of mouse models for human cancer research.”

3. The manuscript acknowledges that spatial omics foundation models lack standardized benchmarking but does not sufficiently elaborate on this critical gap. For histopathology foundation models, tumor-type classification is noted as a common benchmark, but the review does not propose how to evaluate more complex tasks essential for cancer research—such as distinguishing fine-grained tumor microenvironments (e.g., immune-hot vs. immune-excluded regions) or predicting treatment response from spatial patterns. Similarly, for spatial transcriptomics/proteomics models, there is no discussion of reference datasets, gold-standard metrics (beyond clustering or imputation accuracy), or inter-lab reproducibility challenges. This omission is problematic because without agreed-upon benchmarks, progress in the field risks being fragmented and difficult to compare. The review would benefit from proposing a roadmap for community-driven standardization, including multi-scale evaluation tasks and validation against clinical endpoints (e.g., patient survival).

Response: We added a new subsection titled ‘Model benchmarking’ to the ‘Data consideration’ section to address this issue:
“Rapid progress in digital pathology foundation models make rigorous benchmarking essential. Current de facto H&E benchmarks, such as TCGA subtype classification and molecular prediction [16], [17], largely reflect historical data availability rather than a coordinated community-driven effort aimed at biological discovery and translational impact [18]. To close this gap, the field needs community initiatives to curate agreed-upon public datasets and standardize metrics. While clinically grounded datasets and benchmarks are emerging [19], [20], broad institutional momentum remains limited; nonetheless, we anticipate increasing standardization in the coming years.
In the SRO domain the need is even more acute, as limited large-scale datasets and rapidly evolving assays make benchmarking ambiguous. Existing benchmarks mostly target unsupervised tasks (e.g. spatial domain identification) or self-supervised objectives (e.g. imputing missing genes), or rely on expert-annotated datasets that are small and domain-specific [21]. Where labels exist, they are typically intra-tissue (e.g. cell type annotations) rather than cross-sample, and patient-level annotation is scarce. These benchmarks are valuable for research, but to build parallels to the H&E AI models would require clinically

grounded benchmarks built on large-scale patient-level annotations. Organizing consortia to generate clinically annotated, patient-derived spatial omics datasets is essential to establish relevant ground truth and enable clinically meaningful benchmarking."

References

[1] "stDiff: a diffusion model for imputing spatial transcriptomics through single-cell transcriptomics | Briefings in Bioinformatics | Oxford Academic." Accessed: Oct. 02, 2024. [Online]. Available: https://academic.oup.com/bib/article/25/3/bbae171/7646375
[2] X. Li, F. Zhu, and W. Min, "SpaDiT: Diffusion Transformer for Spatial Gene Expression Prediction using scRNA-seq," July 18, 2024, arXiv: arXiv:2407.13182. doi: 10.48550/arXiv.2407.13182.
[3] Q. Jiang and L. Wan, "A physics-informed neural SDE network for learning cellular dynamics from time-series scRNA-seq data," Bioinformatics, vol. 40, no. Supplement_2, pp. ii120–ii127, Sept. 2024, doi: 10.1093/bioinformatics/btae400.
[4] I. K. Boudjelthia, S. Milite, N. E. Kazwini, Y. Huang, A. Sottoriva, and G. Sanguinetti, "NeuroVelo: interpretable learning of temporal cellular dynamics from single-cell data," June 10, 2024, bioRxiv. doi: 10.1101/2023.11.17.567500.
[5] H. Li et al., "Decoding functional cell–cell communication events by multi-view graph learning on spatial transcriptomics," Brief. Bioinform., vol. 24, no. 6, p. bbad359, Nov. 2023, doi: 10.1093/bib/bbad359.
[6] B. Ji, L. Xu, and S. Peng, "SpaCCC: Large language model-based cell-cell communication inference for spatially resolved transcriptomic data," Feb. 23, 2024, bioRxiv. doi: 10.1101/2024.02.21.581369.
[7] C. Zhang, D. Song, C. Huang, A. Swami, and N. V. Chawla, "Heterogeneous Graph Neural Network," in Proceedings of the 25th ACM SIGKDD International Conference on Knowledge Discovery & Data Mining, in KDD '19. New York, NY, USA: Association for Computing Machinery, July 2019, pp. 793–803. doi: 10.1145/3292500.3330961.
[8] C. Zuo, J. Xia, and L. Chen, "Dissecting tumor microenvironment from spatially resolved transcriptomics data by heterogeneous graph learning," Nat. Commun., vol. 15, no. 1, p. 5057, June 2024, doi: 10.1038/s41467-024-49171-7.
[9] "[2206.02647] Scaling Vision Transformers to Gigapixel Images via Hierarchical Self-Supervised Learning." Accessed: Aug. 22, 2025. [Online]. Available: https://arxiv.org/abs/2206.02647
[10] A. F. Pour et al., "Prediction of Outcome from Spatial Protein Profiling of Triple-Negative Breast Cancers," Apr. 18, 2025, bioRxiv. doi: 10.1101/2025.04.18.649541.
[11] R. Ben-Uri et al., "High-dimensional imaging using combinatorial channel multiplexing and deep learning," Nat. Biotechnol., pp. 1–14, Mar. 2025, doi: 10.1038/s41587-025-02585-0.
[12] S. Ayub, H. W. Jackson, A. Selega, and K. R. Campbell, "Multi-view deep learning of highly multiplexed imaging data improves association of cell states with clinical outcomes," Mar. 17, 2025, bioRxiv. doi: 10.1101/2025.03.14.643377.
[13] M. Shaban et al., "A Foundation Model for Spatial Proteomics," June 05, 2025, arXiv: arXiv:2506.03373. doi: 10.48550/arXiv.2506.03373.
[14] E. Haber, A. Deshpande, J. Ma, and S. Krieger, "Unified integration of spatial transcriptomics across platforms," Apr. 21, 2025, bioRxiv. doi: 10.1101/2025.03.31.646238.
[15] T. Shay et al., "Conservation and divergence in the transcriptional programs of the human and mouse immune systems," Proc. Natl. Acad. Sci., vol. 110, no. 8, pp. 2946–2951, Feb. 2013, doi: 10.1073/pnas.1222738110.
[16] R. J. Chen et al., "Towards a general-purpose foundation model for computational pathology," Nat. Med., vol. 30, no. 3, pp. 850–862, Mar. 2024, doi: 10.1038/s41591-024-02857-3.
[17] H. Xu et al., "A whole-slide foundation model for digital pathology from real-world data," Nature, vol. 630, no. 8015, pp. 181–188, June 2024, doi: 10.1038/s41586-024-07441-w.
[18] F. Mahmood, "A benchmarking crisis in biomedical machine learning," Nat. Med., vol. 31, no. 4, pp. 1060–1060, Apr. 2025, doi: 10.1038/s41591-025-03637-3.
[19] G. Campanella et al., "A clinical benchmark of public self-supervised pathology foundation models," Nat. Commun., vol. 16, no. 1, p. 3640, Apr. 2025, doi: 10.1038/s41467-025-58796-1.
[20] A. Zhang, G. Jaume, A. Vaidya, T. Ding, and F. Mahmood, "Accelerating Data Processing and Benchmarking of AI Models for Pathology," Feb. 10, 2025, arXiv: arXiv:2502.06750. doi: 10.48550/arXiv.2502.06750.
[21] R. Zahedi et al., "Deep learning in spatially resolved transcriptomics: a comprehensive technical view," Brief. Bioinform., vol. 25, no. 2, p. bbae082, Mar. 2024, doi: 10.1093/bib/bbae082.

Cookie Preference Center

We use cookies which are necessary to make our site work. We may also use additional cookies to analyze, improve and personalize our content and your digital experience. For more information, see our Cookie Policy.

You may choose not to allow some types of cookies. However, blocking some types may impact your experience of our site and the services we are able to offer. See the different category headings below to find out more or change your settings.

Allow all

Manage Consent Preferences

Strictly Necessary Cookies

Always active

These cookies are necessary for the website to function and cannot be switched off in our systems. They are usually only set in response to actions made by you which amount to a request for services, such as setting your privacy preferences, logging in or filling in forms. You can set your browser to block or alert you about these cookies, but some parts of the site will not then work.

Functional Cookies

These cookies enable the website to provide enhanced functionality and personalisation. They may be set by us or by third party providers whose services we have added to our pages. If you do not allow these cookies then some or all of these services may not function properly.

Performance Cookies

These cookies allow us to count visits and traffic sources so we can measure and improve the performance of our site. They help us to know which pages are the most and least popular and see how visitors move around the site.

Targeting Cookies

These cookies may be set through our site by our advertising partners. They may be used by those companies to build a profile of your interests and show you relevant adverts on other sites. If you do not allow these cookies, you will experience less targeted advertising.

Cookie List

Clear

Apply Cancel

Consent Leg.Interest

Confirm my choices

Powered by onetrust
