# Supplementary material for: Emerging AI approaches for cancer spatial omics
Source: Gigascience. 2025 Oct 16;14:giaf128. doi: 10.1093/gigascience/giaf128 (PMC12612624; doi:10.1093/gigascience/giaf128)
Supplement: giaf128_Reviewer_1_Report_Original_Submission — Carolina Wählby -- 7/11/2025 [file giaf128_reviewer_1_report_original_submission.pdf]

Reviewer Report

Title: Emerging AI Approaches for Cancer Spatial Omics

Version: Original Submission **Date:** 7/11/2025

Reviewer name: Carolina WÃ¤hlby

Reviewer Comments to Author:

This is a very nice review providing a new and original angle to the present stat e of combining AI-based analysis approaches with spatially resolved omics methods (brrimarily proteomics and transcriptomics). The review also points to new directions of development, and is truly inspirational in its outlooks and future perspectives, which is appreciated. The text is well-written and easy to follow, though at points a bit brief in methods descriptions, as can be expected from this type of broad review paper.I was missing references in the introduction, feeling that many of the statements would have benefitted from pointing to e.g. other review papers in the field. Adding more references to the introduction would improve the text and also make the review more useful for an audience without expert knowledge in spatially resolved omics.Abbreviations are thoroughly described apart from 'patient IRB', which should be defined, or perhaps re-phrased to not use an abbreviation.

Level of Interest

Please indicate how interesting you found the manuscript: Choose an item.

Quality of Written English

Please indicate the quality of language in the manuscript: Choose an item.

Declaration of Competing Interests

Please complete a declaration of competing interests, considering the following questions:

- Have you in the past five years received reimbursements, fees, funding, or salary from an organisation that may in any way gain or lose financially from the publication of this manuscript, either now or in the future?
- Do you hold any stocks or shares in an organisation that may in any way gain or lose financially from the publication of this manuscript, either now or in the future?
- Do you hold or are you currently applying for any patents relating to the content of the manuscript?
- Have you received reimbursements, fees, funding, or salary from an organization that holds or has applied for patents relating to the content of the manuscript?
- Do you have any other financial competing interests?
- Do you have any non-financial competing interests in relation to this paper?

If you can answer no to all of the above, write 'I declare that I have no competing interests' below. If your reply is yes to any, please give details below.

I declare that I have no competing interests

I agree to the open peer review policy of the journal. I understand that my name will be included on my report to the authors and, if the manuscript is accepted for publication, my named report including any attachments I upload will be posted on the website along with the authors' responses. I agree for my report to be made available under an Open Access Creative Commons CC-BY license (http://creativecommons.org/licenses/by/4.0/). I understand that any comments which I do not wish to be included in my named report can be included as confidential comments to the editors, which will not be published.

Choose an item.

To further support our reviewers, we have joined with Publons, where you can gain additional credit to further highlight your hard work (see: https://publons.com/journal/530/gigascience). On publication of this paper, your review will be automatically added to Publons, you can then choose whether or not to claim your Publons credit. I understand this statement.

Yes Choose an item.
