# Supplementary material for: Emerging AI approaches for cancer spatial omics
Source: Gigascience. 2025 Oct 16;14:giaf128. doi: 10.1093/gigascience/giaf128 (PMC12612624; doi:10.1093/gigascience/giaf128)
Supplement: giaf128_Reviewer_2_Report_Original_Submission — Yingcheng Wu -- 8/8/2025 [file giaf128_reviewer_2_report_original_submission.pdf]

Reviewer Report

Title: Emerging AI Approaches for Cancer Spatial Omics

Version: Original Submission **Date:** 8/8/2025

Reviewer name: Yingcheng Wu

Reviewer Comments to Author:

1. The manuscript outlines three emerging paradigms (data-driven, constraint-based, and mechanistic spatial AI) but lacks a rigorous critical analysis of their practical limitations in cancer research. For data-driven foundation models, while post-hoc interpretation methods (e.g., SHAP, LIME) are mentioned, the review does not sufficiently address their inherent weaknessesâ€”such as the inability to establish causal relationships or the risk of overfitting to batch effects in heterogeneous spatial omics datasets. Similarly, constraint-based models (e.g., diffusion models) are noted for integrating spatial hierarchies, but there is little discussion of how their performance scales with high-dimensional spatial proteomics data (e.g., >1000 markers) or their susceptibility to artifacts from variable antibody panels. Mechanistic models like PINNs are highlighted, yet the manuscript overlooks critical barriers such as the scarcity of longitudinal spatial data in clinical cancer samples, which limits their training and validation. A more balanced assessment of these limitations is essential for readers to gauge the current translational readiness of each paradigm.2. The review emphasizes the importance of multi-modal data integration (e.g., H&E, spatial transcriptomics, proteomics) but fails to address key technical bottlenecks in achieving robust integration. For instance, while methods like SpatialGlue and OmiCLIP are cited, there is little discussion of how to resolve fundamental inconsistencies: varying spatial resolutions (0.25-100 microns across technologies), platform-specific noise (e.g., dropout in spatial RNA), and divergent marker panels in proteomics. This gap hinders understanding of the current feasibility of holistic spatial profiling. Additionally, despite noting mouse models as critical for mechanistic validation, the manuscript underdeveloped the challenge of cross-species translation. While BrainAlign and Nicheformer are mentioned, there is insufficient analysis of how species-specific differences in tissue architecture (e.g., immune microenvironment organization) or gene expression dynamics might invalidate model transferability. Without addressing these, the review risks overstating the practical utility of current integration frameworks.3. The manuscript acknowledges that spatial omics foundation models lack standardized benchmarking but does not sufficiently elaborate on this critical gap. For histopathology foundation models, tumor-type classification is noted as a common benchmark, but the review does not propose how to evaluate more complex tasks essential for cancer researchâ€”such as distinguishing fine-grained tumor microenvironments (e.g., immune-hot vs. immune-excluded regions) or predicting treatment response from spatial patterns. Similarly, for spatial transcriptomics/proteomics models, there is no discussion of reference datasets, gold-standard metrics (beyond clustering or imputation accuracy), or inter-lab reproducibility challenges. This omission is problematic because without agreed-upon benchmarks, progress in the field risks being fragmented and difficult to compare. The review would benefit from proposing a roadmap for community-driven standardization, including multi-scale evaluation tasks and validation against clinical endpoints (e.g., patient survival).

Level of Interest

Please indicate how interesting you found the manuscript: Choose an item.

Quality of Written English

Please indicate the quality of language in the manuscript: Choose an item.

Declaration of Competing Interests

Please complete a declaration of competing interests, considering the following questions:

- Have you in the past five years received reimbursements, fees, funding, or salary from an organisation that may in any way gain or lose financially from the publication of this manuscript, either now or in the future?
- Do you hold any stocks or shares in an organisation that may in any way gain or lose financially from the publication of this manuscript, either now or in the future?
- Do you hold or are you currently applying for any patents relating to the content of the manuscript?
- Have you received reimbursements, fees, funding, or salary from an organization that holds or has applied for patents relating to the content of the manuscript?
- Do you have any other financial competing interests?
- Do you have any non-financial competing interests in relation to this paper?

If you can answer no to all of the above, write 'I declare that I have no competing interests' below. If your reply is yes to any, please give details below.

I declare that I have no competing interests'

I agree to the open peer review policy of the journal. I understand that my name will be included on my report to the authors and, if the manuscript is accepted for publication, my named report including any attachments I upload will be posted on the website along with the authors' responses. I agree for my report to be made available under an Open Access Creative Commons CC-BY license (http://creativecommons.org/licenses/by/4.0/). I understand that any comments which I do not wish to be included in my named report can be included as confidential comments to the editors, which will not be published.

Choose an item.

To further support our reviewers, we have joined with Publons, where you can gain additional credit to further highlight your hard work (see: https://publons.com/journal/530/gigascience). On publication of

this paper, your review will be automatically added to Publons, you can then choose whether or not to claim your Publons credit. I understand this statement.

Yes Choose an item.
